# Quantitative ethnoveterinary study on plant resource utilization by indigenous communities in high-altitude regions

**DOI:** 10.3389/fvets.2022.944046

**Published:** 2022-10-06

**Authors:** Shiekh Marifatul Haq, Umer Yaqoob, Muhammad Majeed, Muhammad Shoaib Amjad, Musheerul Hassan, Riyaz Ahmad, Muhammad Waheed, Rainer Willi Bussmann, Eduardo Soares Calixto, Jarosław Proćków, José M. Pérez de la Lastra, Antonio Morales-de la Nuez

**Affiliations:** ^1^Clybay Research Private Limited, Bangalore, India; ^2^Department of Ethnobotany, Institute of Botany, Ilia State University, Tbilisi, Georgia; ^3^Zonal Educational Office, Shopian, India; ^4^Department of Botany, University of Gujrat, Gujrat, Pakistan; ^5^Department of Botany, Women University of Azad Jammu and Kashmir, Bagh, Pakistan; ^6^School of Geography, Earth and Environmental Science, University of Birmingham, Birmingham, United Kingdom; ^7^National Center for Wildlife, Riyadh, Saudi Arabia; ^8^Department of Botany, University of Okara, Punjab, Pakistan; ^9^Department of Entomology and Nematology, Institute of Food and Agricultural Sciences, University of Florida, Gainesville, FL, United States; ^10^Department of Plant Biology, Institute of Environmental Biology, Wrocław University of Environmental and Life Sciences, Wrocław, Poland; ^11^Biotechnology of Macromolecules, Institute of Natural Products and Agrobiology, CSIC-Spanish Research Council, Madrid, Spain

**Keywords:** medicinal, magical, fodder preparations, livelihood, ethnic communities, Himalayas

## Abstract

For millennia, ethnic knowledge has been intricately tied to local biodiversity and woven into the fabric of rural communities. Growing scientific evidence suggests that merging ethnic knowledge with new scientific findings can lead to socially acceptable and environmentally friendly approaches essential for the long-term prosperity of local communities. In the high-altitude region, where livestock raising is a key income source, and plant-based utilization for ethno-veterinary practices is widely practiced. In this context, this study was conducted with the aim of documenting the ethno-veterinary use of plant resources in different bio-geographical regions of Jammu and Kashmir's Himalayas (J & KH). Semi-structured interviews and group discussions were used to collect information. Principal component analysis (PCA) and Pearson correlation were conducted to analyze the data. We documented 148 species from 53 families that locals used for various purposes: medicine, fodder, tonic, antidote, magic, and also used to protect themselves from ectoparasite such as *Pediculus humanus capitis* by the local inhabitants. There were significant differences in the relative usage of plant resources across the three biogeographic regions. Comparatively, the highest number (41%) of plant species were used for ethnoveterinary in the Jammu region, while the lowest number (28%) of species were used in Kashmir. Across the regions, Kashmir and Jammu had the highest level of species similarity (17%), while Jammu and Ladakh had the lowest (1%). A cross-regional assessment of plant resources revealed that 18% of plants were shared among the regions. The reported use of *Amaranthus blitum, Morus alba, Ficus palmata, Vitex negundo, Juniperus semiglobosa, Ulmus wallichiana*, and *Rumex nepalensis* are novel for the ethno-veterinary uses of this part of the Himalayan region. The various dry unique traditional fodder preparations (*gaaslov, gass khor, pan baath, kaandbaath, Lovgooad, Karb*, and *Phungma*) from plant resources are reported for the first time from the Himalayan region and can be ascribed to the novelty of this study. Plant resources were not only a source of fodder and medicine but also presented themselves as an opportunity for livelihood generation. Therefore, our findings bridge the knowledge gap by documenting key ethnoveterinary applications of native plant species from the study region that are used to cure livestock diseases and disorders by the mountain inhabitants.

## Introduction

Ethnoveterinary science is a vast field that encompasses people's perspectives on veterinary healthcare, including their beliefs, skills, knowledge, and practices (1). Medicinal herbs, which have been used to treat animal ailments for centuries, play an essential role in local health practices. Allopathic modalities are usually unavailable in rural veterinary care, especially in developing countries; therefore, phototherapeutics are frequently used as the primary type of therapy (2). As a result, pastoral and agricultural communities that rely significantly on cattle for their income and food security value local knowledge of ecological resources for veterinary care (3). On the other hand, traditional ethnoveterinary knowledge is mostly passed down orally from generation to generation (i.e., in the form of traditional remedies, drawings, stories, poems, proverbs, folk myths, and songs). Because of the nature of oral transmission, this type of local knowledge is vulnerable and endangered, necessitating its recording and documentation (4). Moreover, as people's tastes alter in response to significant socioeconomic transitions, which are coordinated with environmental changes and technological developments.

Plant-based ethnoveterinary medicine is widely used in the Himalayan region, where livestock rearing is a major source of income. In comparison to western allopathic pharmaceuticals, traditional herbal remedies provide effective, inexpensive therapies, and widely accessible treatments (5–7). This ethnic knowledge is inextricably tied to the local biodiversity and has been woven into the rural communities' fabric for centuries. Consequently, herbal treatments are to the indigenous Himalayan therapeutic practices (9, 10). The documentation of this altruistic folk knowledge is essential now that the Nagoya Protocol has been ratified to preserve cultural heritage (5). Growing scientific data demonstrates that combining ethnic knowledge with new scientific discoveries might result in socially acceptable and environmentally benign techniques that are critical for the preservation of local communities (8).

Assessing the monetary values of plant-based ethno-veterinary in relation to the rising cost of cattle breeding and care is an intriguing topic (5, 11). Furthermore, ethno-veterinary medicine is particularly active and versatile in that it can cure a variety of cattle ailments, is widely accessible in remote locations, and is less expensive than manufactured medications (12). The interaction between humans, plants, and animals has existed since the beginning of time when humans discovered natural resources to meet their requirements, including respite from personal maladies for themselves as well as those of their domestic animals (13, 14). The researchers have worked extensively on ethno-medicinal plant applications for human health (15, 16); however, there have been very few investigations into ethno-veterinary applications of native herbs in the region, indicating a large knowledge gap.

The erstwhile state of Jammu and Kashmir has a rich history of plant resources that could help in identifying novel and potential sources of medicines, fodder, and other plant products. Many studies have been carried out to document the associated traditional knowledge. We investigated the ethno-veterinary use of plant species in each biogeographic region to discover how social, economic, cultural, religious, and climatic factors influenced plant usage patterns and compositions. The fieldwork's main goals were to [1] gain a thorough grasp of ethnomedicinal, cultural, and magical aspects of plant diversity in the region and [2] assess how fodder plants could improve livelihoods and aid in poverty alleviation. Furthermore, this study aims to present comprehensive data on the use of plants as medicinal and ethnoveterinary practices by different indigenous ethnic communities in Jammu and Kashmir's Himalayas.

## Materials and methods

### Study area

**Figure 1:**
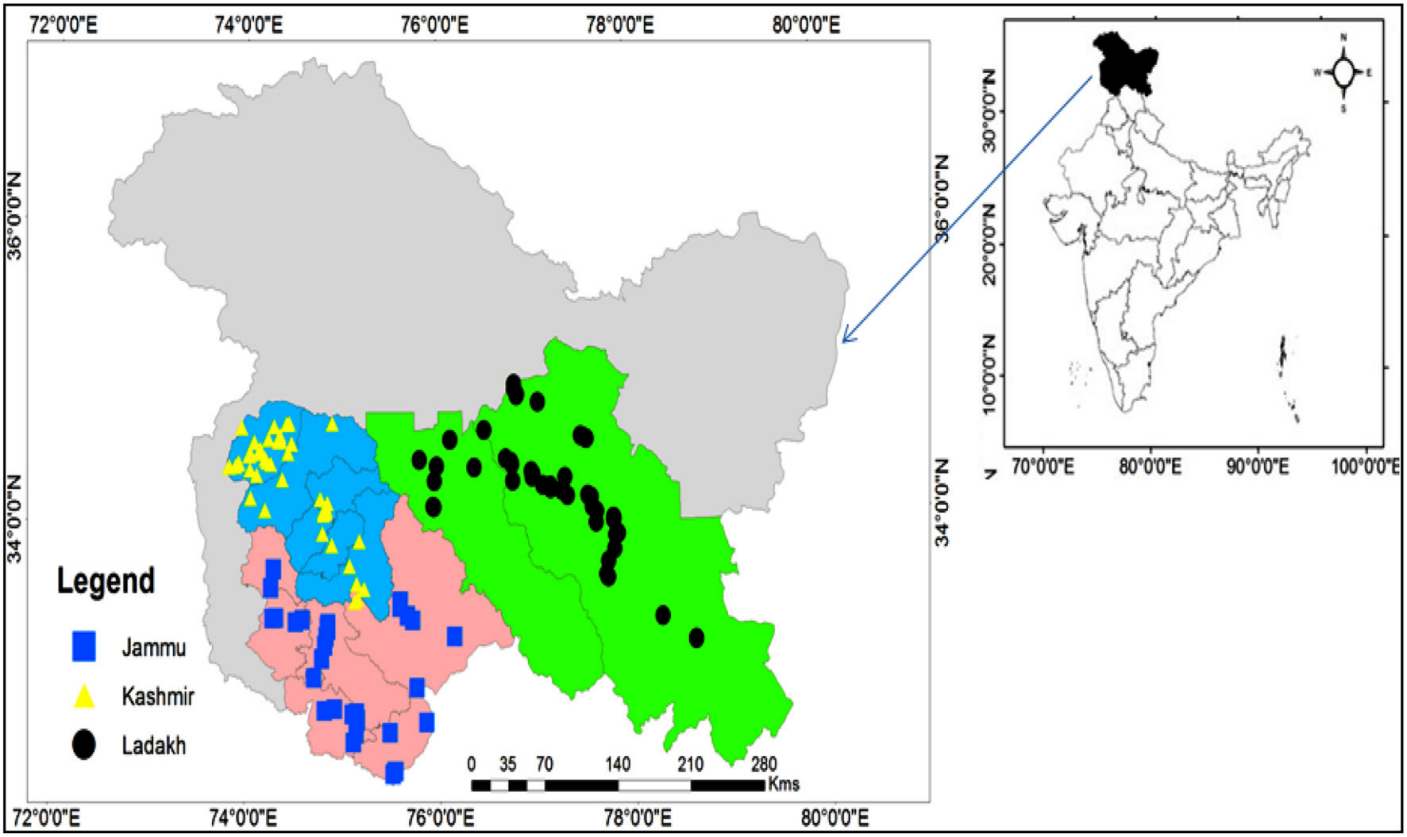
Map of the Jammu and Kashmir (J&K) and point showing the sampling sites in erstwhile princely state of Jammu and Kashmir. The background colors in the map of different regions were pale yellow color for Ladakh, sky blue color for Kashmir, light pink color for Jammu region.

Three biogeographic regions (Kashmir, Ladakh, and Jammu) (Figure 1) of the erstwhile state (Jammu and Kashmir) divided into two union territories (Ladakh and Jammu and Kashmir) were chosen for this study, each with its own faiths, languages, ethnicity, and terrain (Table 1). Jammu and Kashmir is located to the west of Ladakh and north of Himachal Pradesh (India). The realm is divided into two provinces: Jammu and Kashmir. Ladakh is ruled by two countries—India and China—and shares its borders with Gilgit-Baltistan (Pakistan) to the northwest, Himachal Pradesh to the west, and Tibet to the north. The erstwhile state of Jammu and Kashmir was home to a rich ethnic and cultural diversity. The climate varies across the state; in Jammu province, the annual minimum temperature is 17.9°C, and the maximum 29.6°C with an annual rainfall of 1,331 mm, whereas in Kashmir province, the annual minimum temperature observed is 7.3°C and the maximum 19.7° C with an average rainfall of 710 mm. In the Ladakh region, which is a cold desert, the annual minimum temperature is 1.2°C, and the maximum is 12.3°C with annual precipitation of 102 mm. The socioeconomic situation in the study area is diverse, with agriculture and allied activities being the primary sources of income. The majority of the population comprises farmers, while others are government employees and daily workers. The Gujjar and Bakharwal communities are predominantly associated with livestock, whereas the Kashmiri, Pahari, and Dogra communities are predominantly associated with agriculture and horticulture. People living in remote and hilly areas have a strong cultural belief in the traditional medicinal system.

**Table 1:** Demographic status of the respondents from the study area.

**Demographic features**	**Total informants (Percentage)**	**Biogeographic regions**			**(Linguistic) Ethnic groups**						
					**Kashmiri**	**Pahari**	**Bakharwal**	**Gujjar**	**Dogra**	**Balti**	**Brokpa**
		**Jammu**	**Kashmir**	**Ladakh**							
**Respondents**	229	77 (33.62%)	91 (39.73%)	61 (26.63%)	45 (19.65%)	36 (15.72%)	29 (12.66%)	35 (15.28%)	23 (10.04%)	29 (12.66%)	32 (13.97%)
**Gender**		
Male	146 (63%)	41 (17.90%)	65 (28.38%)	40 (17.46%)	35 (15.28%)	20 (8.73%)	21 (9.17%)	20 (8.73%)	10 (4.36%)	18 (7.86%)	22 (9.60%)
Female	83 (36.24%)	36 (15.72%)	26 (11.35%)	21 (9.17%)	10 (4.36%)	16 (6.98%)	8 (3.49%)	15 (6.55%)	13 (5.67%)	11 (4.80%)	10 (4.36%)
Original Languag					Kashmiri	Pahari	Gujari	Gujari	Dogri	Balti	Brokpa
Age range					(27–75)	(27–75)	(27–75)	(27–75)	(27–75)	(27–75)	(27–75)
Religion		Islam Hinduism Sikhism	Islam Hinduism		Shia and suni Islam Sikhism	Shia and suni Islam Hinduism	Suni Islam	Suni Islam Hinduism	Hinduism	Shia Islam Buddhism	Suni Islam Buddhism
Topography					Plan areas	Hilly areas	Hilly areas	Hilly areas	Plan areas	Hilly areas	Hilly areas
Livelihood source					Agriculture Horticulture	Agriculture	Pastoralism	Agriculture Pastoralism	Horticulture Agriculture	Cattle rearing Horticulture	Cattle rearing Wage labor

This study is based on a field survey conducted between 2019 and 2021. We investigated the indigenous communities of Dogra, Balti, Brokpa, Bakharwal, Gujjar, Kashmiri, and Pahari. It is worth noting that the Dogra community is largely found in the Jammu province, whereas the Balti and Brokpa are exclusive to Ladakh. A total of 229 respondents were selected, of whom 146 were male and 83 were female (Table 1). All selected ethnic communities had different languages and faiths and were interviewed numerous times. The methodology was based on interviews (*n* = 194) and group discussions (*n* = 35) to enlist the taxa with ethnoveterinary values (17, 18). The information on their age, gender, profession, and education was also documented. As participants mentioned, local plant names, parts used, valuable key species, collection season and availability status, and preferred species for utilization were all documented. All the interviews were carried out in the local language (by employing translator services) after receiving informed consent in accordance with the Ethics Code (International Society of Ethnobiology) (19). During plant collection, we sought the assistance of one of the knowledgeable informants for the confirmation of the species to obtain an error-free herbarium by using proper coding. A pictorial field guide (20) or live plants were shown to the informants to produce the local names. Furthermore, there were some conflicts in the local name, which were resolved by organizing group discussions. For the scientific validation of the collected plant taxa, we cross-checked with the help of taxonomists at the Center of Biodiversity and Taxonomy, University of Kashmir, Srinagar (Jammu and Kashmir). To authenticate the correct names of the plant species, Plants of the World Online 2019 (http://www.plantsoftheworldonline.org/) was used. The voucher specimens were deposited at the KASH herbarium.

### Data analysis

Principal component analysis (PCA) (16) was used to determine hypothetical variables (components) that explain as much of the variance in our multidimensional data as possible in associational analyses between different ethnic groups and plant compositions. We calculated the singular value decomposition of the (centered and possibly scaled) data matrix using a matrix of plant species presence/absence in each of the three regions analyzed. R Studio 4.0.1 was used to perform the PCA. Using PCA, we could determine the relationship between each species or group of species and each area and ethnic group. The Bioinformatics & Evolutionary Genomics software (21) was used to produce the Venn diagram to conduct cross-cultural comparisons between regions (http://bioinformatics.psb.ugent.be/cgi-bin/liste/Venn/calculatevenn.htpl). The dendrogram depicts species distribution using presence/absence data, as well as the clustering approach groups species that share the same provisioning services. The Sorensen's (Bray-Curtis) distance (22) was used with the Past program ver. 3.14 to detect significant differences among various supplying services and plant resemblances (16). Finally, we used the Pearson method to calculate the correlation coefficient between ethnic groups (Pahari, Gujjar, Kashmiri, Dogra, Bakharwal, Brokpa, and Balti) and regions. The results were plotted in a correlogram (23) with the “corrplot” package (24).

## Results and discussions

### General plant compositions and distribution patterns

Local people in the three bio-geographical regions employed a total of 148 plant species from 53 families that locals used for various purposes: medicine, fodder, tonic, antidote, and magic, as well as using them to protect not just themselves against ectoparasites but also animals (Supplementary Table 1). The current study's species numbers are higher than earlier fragmented ethno-veterinary studies from the same region, which focused mainly on one or a few districts in Jammu and Kashmir [e.g., (25–30)]. By comparing the presently documented species with the allied areas (31–35), it became evident that J & K, in the Western Himalayan region, are more adept at utilizing plant resources for ethno-veterinary purposes than these parts of the Himalayas.

**Figure 2:**
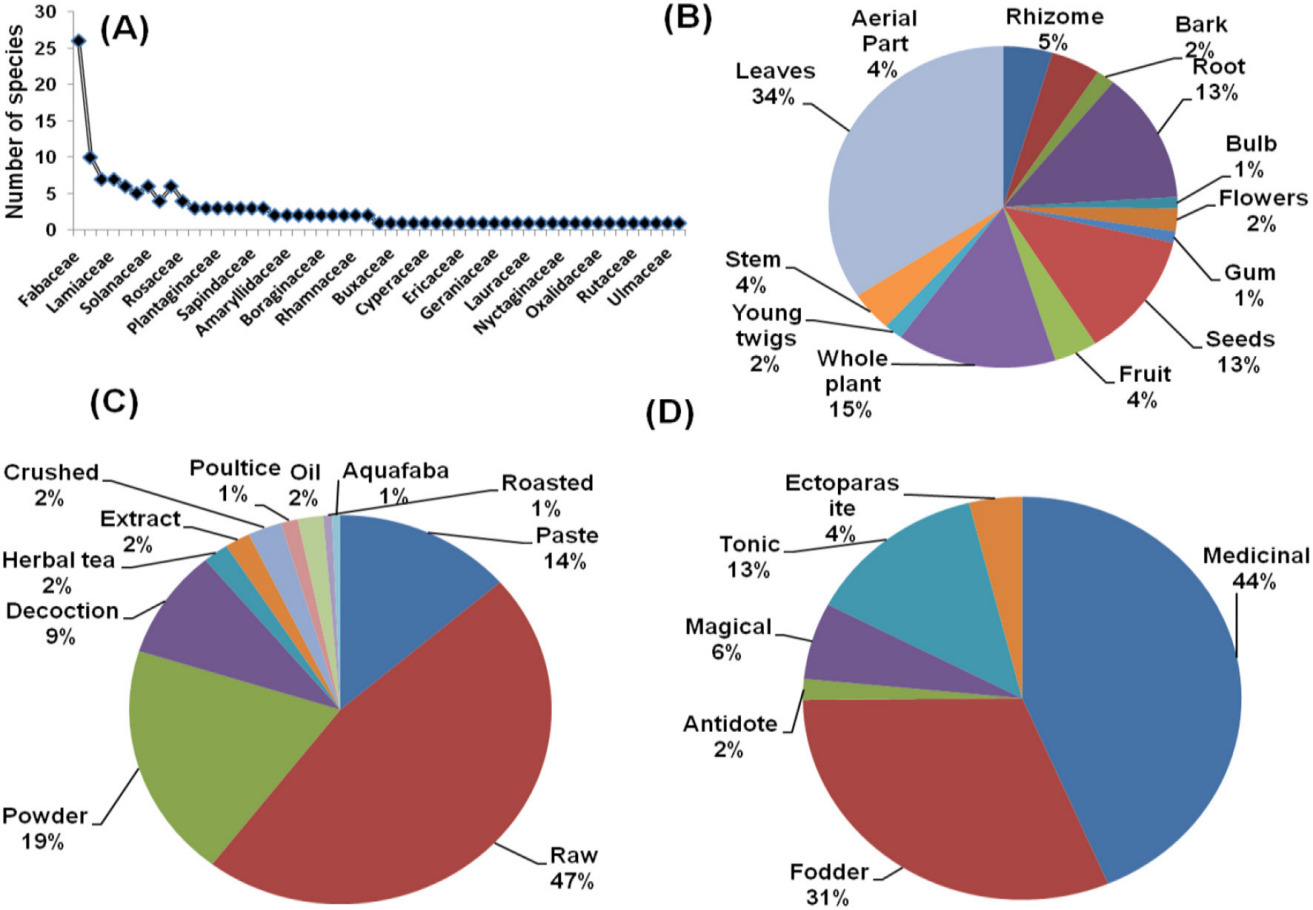
**(A)** Species family relationship **(B)** part used **(C)** form of preparation **(D)** ethno-veterinary usage of plants from three bio geographic regions.

The species distribution across 53 families was disproportionate (Figure 2A illustrates the species-family relationship). The Fabaceae family had the most species (18%), followed by the Asteraceae (7%), Brassicaceae (5%), Lamiaceae (5%), and Apiaceae (4% species). Various ethno-veterinary studies produce similar findings (28, 34, 36). Many researchers have also identified the Asteraceae, Brassicaceae, and Lamiaceae families as the most representative of the Himalayan regions (16, 26–29, 32, 33, 37), demonstrating the family's great adaptability in many climates and geographies (38). This study found unequal family distribution patterns with 44 monotypic families, which is consistent with prior ethno-veterinary studies from various Himalayan locations (17, 34, 39).

Different parts of plants were documented for indigenous usage with a significant difference (χ2 = 70.587, df = 13, *p* < 0.001) between their uses. In the current investigation, the most frequently used plant parts were leaves (34%), followed by the whole plant (15%) and roots (13%) (see Figure 2B). Similarly, raw (47%) was the most common form of preparation, followed by powder (19%) and paste (14%) (Figure 2C). Regarding the use category, the medicinal application of plant species received the highest priority from local participants (44 %), followed by fodder (31 %) and tonic (13 %) (Figure 2D). Our findings are consistent with the ethno-veterinary studies conducted in several Himalayan and other regions (28, 34, 40–46).

### Comparative plant composition and usage

The Indian Himalayan state of Jammu and Kashmir is a large biological paradise that spans three biogeographic zones: the Trans-Himalayan zone of Ladakh, the Northwest Himalaya of Kashmir, and the Western Himalaya of Jammu (15). From the plains of Jammu (300 m), it has a captivating habitat diversity across Kashmir's mountainous terrain (1,600–4,250 m), Ladakh's cold desert (> 3,000 m) to the summits of Nanga Parbat (8,126 m) and K2 (8,611 m); the undivided state has an equally rich biota and has been appropriately dubbed a “biomass state.” (16, 17). From the three regions of J & K state – Jammu, Kashmir, and Ladakh – 5,056 taxa (species, subspecies, and varieties) of angiosperm flora have been reported. There are 4,778 species and 278 subspecies/varieties in these taxa, which are divided into 1,306 genera and 180 families. There were significant differences (χ2 = 87.631, df = 6, *p* < 0.001) in the relative usage of plant resources in each biogeographic region. Out of the total species, 41 % were reported to be used in the Jammu region, while 31% were used in Ladakh. However, a minimum of 28% were used for different ethnoveterinary usage by local inhabitants in the Kashmir region. The detailed region-wise plant composition and ethnoveterinary usage is discussed below.

**Figure 3:**
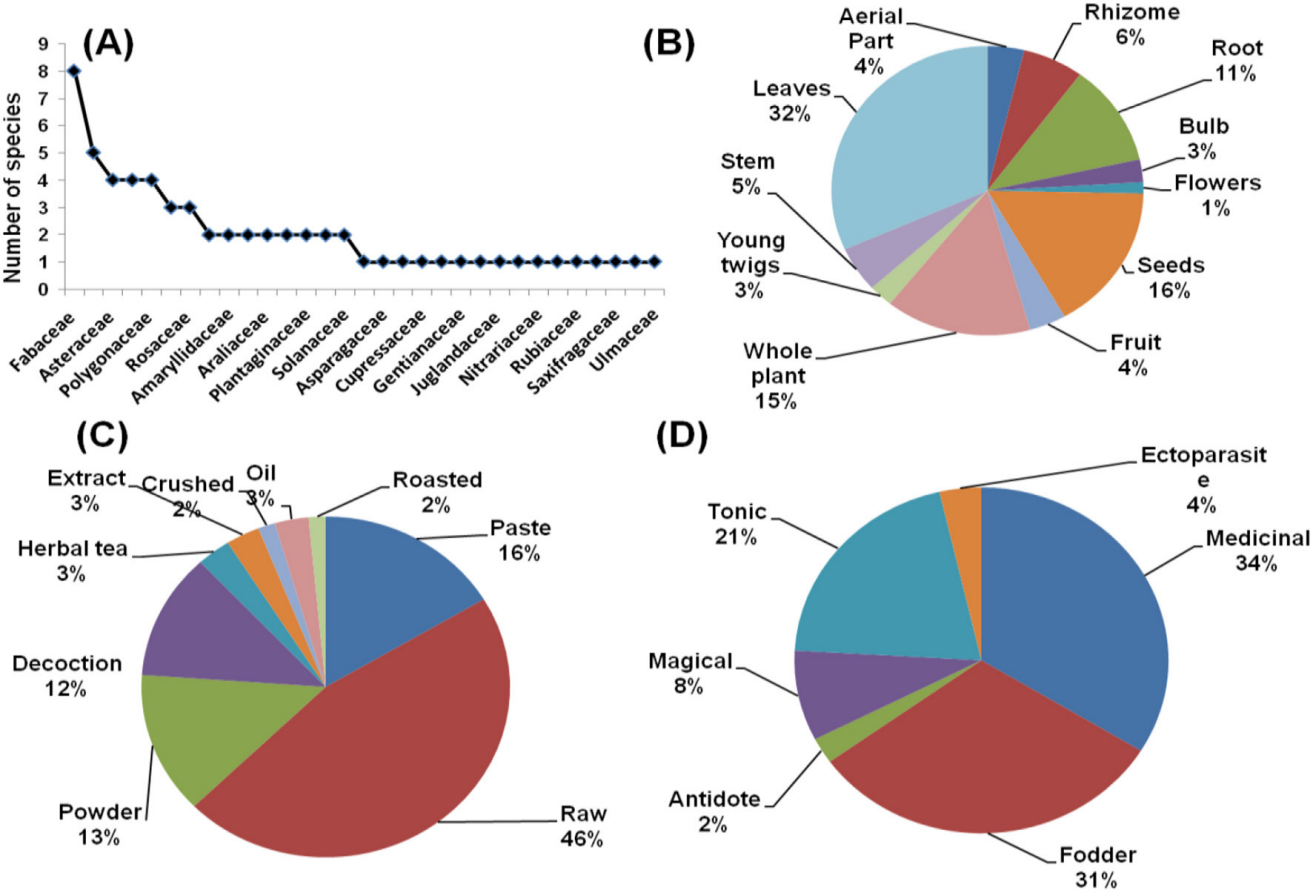
**(A)** Species family relationship **(B)** part used **(C)** form of preparation **(D)** ethno-veterinary usage of plants from Kashmir region.

In the Kashmir region, a total of 64 plant species from 31 families were documented for ethno-veterinary usage. The Fabaceae family had the most species (13%), followed by Poaceae (8%), Asteraceae, and Brassicaceae (6% of species each). Figure 3A depicts the species–family relationship of the recorded plants from the region. The dominance of these families could be attributed to suitable habitats, favorable environmental conditions for the growth of the species, and more interactions of local communities with them (17). The most frequently used plant parts were leaves (32%), followed by whole plants (15%), and roots (11%) (Figure 3B). Furthermore, using green leaves and aerial parts is regarded as safe and sustainable for fodder. Jan et al. (47) also report that fresh and dried leaves are reported as the most used parts of the Gulmarg Kashmir Himalaya. In this study, raw (46%) was the most common form of preparation, followed by paste (16%) and powder (13%) (Figure 3C). The highest priority of local people was the medicinal use of plant species (34% of responses), followed by fodder (31%) and tonic (21%) (Figure 3D, Supplementary Table 1). A similar usage pattern was reported by Kumar and Bharati (48) in the ethno-veterinary study from India.

**Figure 4:**
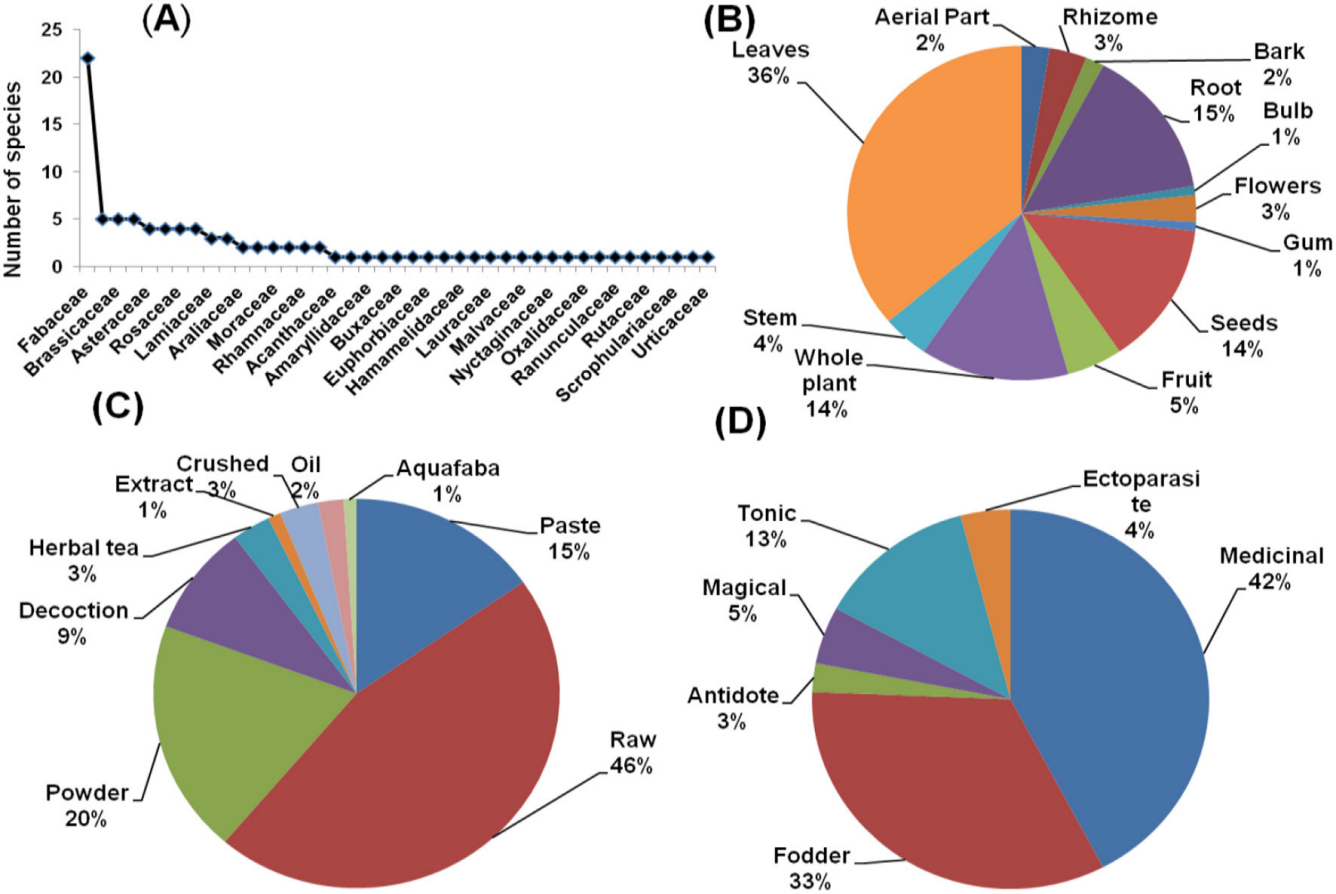
**(A)** Species family relationship **(B)** part used **(C)** form of preparation **(D)** ethno-veterinary usage of plants from Jammu region.

Jammu showed 96 plant species belonging to 41 families that were employed for ethno-veterinary usage. The most species were found in the Fabaceae family (23 percent), followed by Brassicaceae and Asteraceae (5 percent species each). The species–family relationship of the region's reported plants is depicted in Figure 4A. Our results are similar to Bhatti et al. (34), who report Fabaceae as the dominant family used for ethno-veterinary practices. The most frequently used plant parts were leaves (36%), followed by roots (15%) and whole plants (14%) (Figure 4B). Shah et al. (49) and Wani et al. (50) reported the same from the Rajouri-Poonch districts of Jammu. In this study, raw (46%) was the most common form of preparation, followed by powder (20%) and paste (15%) (see Figure 4C). Silambarasan and Ayyanar (51) also reported the same results from the Eastern Ghats, India. The highest priority of local people was the medicinal use of plant species (42% of responses), followed by fodder (33%) and tonic (13%) (Figure 4D). A similar usage pattern was reported by Haq and Singh (17) from District Reasi, J & K, and Patel et al. (36) from India.

**Figure 5:**
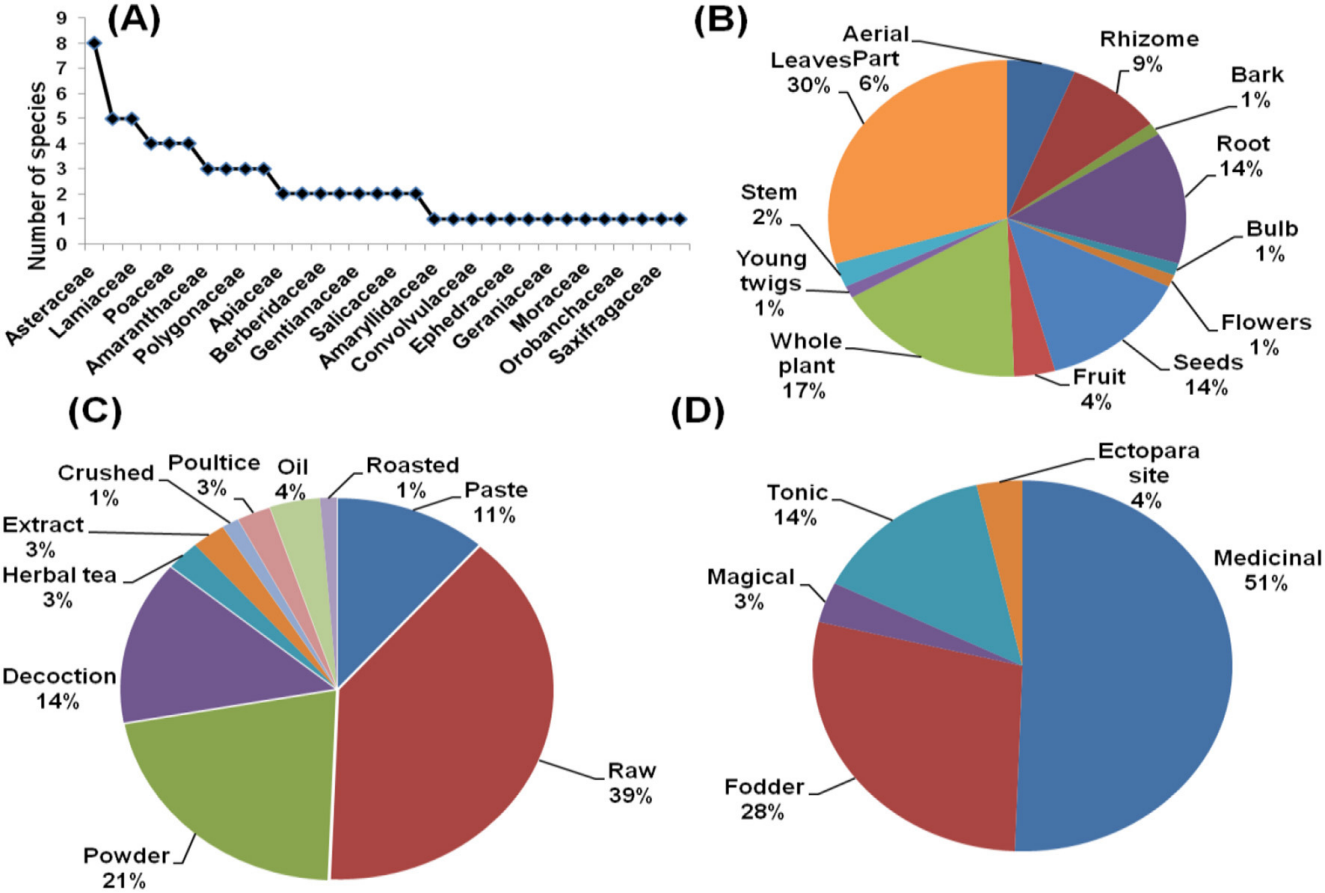
**(A)** Species family relationship **(B)** part used **(C)** form of preparation **(D)** ethno-veterinary usage of plants from Ladakh region.

We reported a total of 72 plant species belonging to 32 families from the Ladakh region. Asteraceae (11% of species) was the most common family, followed by Lamiaceae (7% of species). Figure 5A depicts the species–family relationship of the region's reported plants, which is consistent with previous work showing similar families predominate in Ladakh (16). The most frequently used plant parts were leaves (30%), followed by whole plants (17%) and roots (14%) (see Figure 5B). Ahmad et al. (52) also reported leaves as the most frequently used plant part for the preparation of medicine and fodder for animals. In this study, raw (39%) was the most common form of preparation, followed by powder (21%) and decoction (14%) (Figure 5C). Similar common forms of preparation were reported by Devithakur et al. (53) from the Himalayas. The highest priority of local people was the medicinal use of plant species (51% of responses), followed by fodder (28%) and tonic (14%) (Figure 5D). Murthy et al. (54) also reported similar use of plant resources for ethno-veterinary practices by the Koyas ethnic group in India and Shoaib et al. (39) from the Pakistan Himalayas.

### Seasonal usage of plant resources

**Figure 6:**
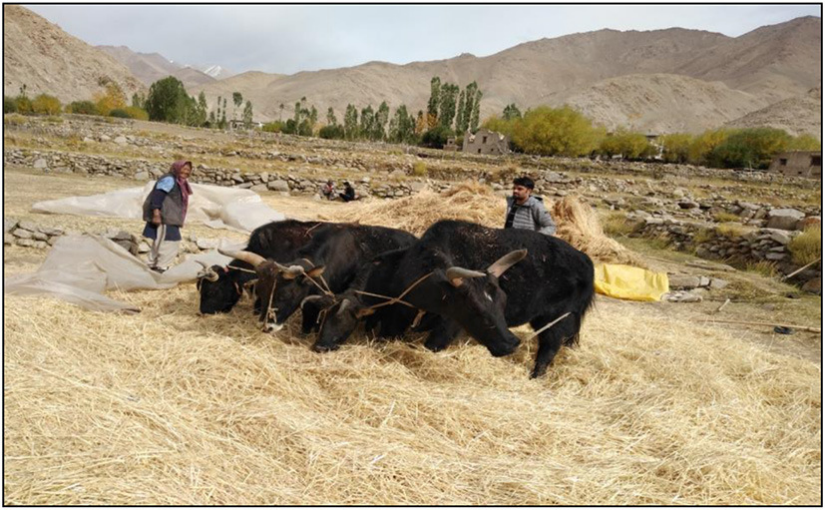
The author is assisting locals in the preparation of fodder locally called as (*Phungma*) from *Hordeum vulgare* in the Ladakh region.

Seasonality influenced how different plant resources were used. Because of their abundance and availability, fresh green plants were favored as fodder in the summer. However, during the summer and autumn seasons, the surplus plant materials were dried and packed into little packs known as *gaaslov, gass khor, pan baath, kaandbaath, and Lovgooad;* they were stored at particular places in heaps called *Thaipar, Lov Kath, Gaaskath*, and *Pan kath* in Kashmir. These are conical or pyramidal; in Jammu, these heaps are known as *Kunnu, Phawara*, and *Gaadhi*and, while in Ladakh, they are called *laptsey*. The major plants stored as fodder for winter in Kashmir were *Oryza sativa* and *Zea mays*. The various species of *Trifolium*, along with *Cynodon dactylon*, formed a unique fodder preparation called *Lov*, which was also used during winter. The seeds of *Brassica campestris* were used to extract oil, and the residue obtained was used as a very nutritive feed for cattle, locally called *Kaj*. In Jammu, the major plant species used as fodder in the winter season were *Triticum aestivum, Avena sativa, Medicago falcata*, and *Hordeum vulgare*, locally called *poaoo, janni, saridha, and jaoon*. The unique fodder preparation was a combination of *Trifolium* species, along with *Cynodon dactylon* and *Zea mays*, locally called *Karb*and *Gaadhi*. The fresh twigs of tree species such as *Grewia optiva, Mallotus philippensis*, and *Aesculus indica* were also dried in the summer; they are locally called *padha* or *parha* and are also used as fodder in the winter season. In Ladakh, the most important plant species used as fodder during harsh winters were *Avena sativa* and *Melilotus officinalis*, locally known as *yukportsua* and *bugsukrtsua*, respectively, and when stored in bales, they were called *Lambu Rtsua*. The crop residues containing mainly the straw of plants such as *Hordeum vulgare* and *Triticum aestivum* constitute a unique fodder preparation called *phungma* (Figure 6) and are also used in winter. The straw of *Fagopyrum esculentum* was also stored in bales locally known as *sokhma*. Leaves of plants were also collected and stored for winter, which is locally known as *skambolunga*. Similar preparations were reported by Jayakumar et al. (55) from Southern India, and Alawa et al. (56) also reported a combination of ingredients used by the traditional ethno-veterinary practices from Nigeria.

### Novel species with ethno-veterinary applications

**Figure 7:**
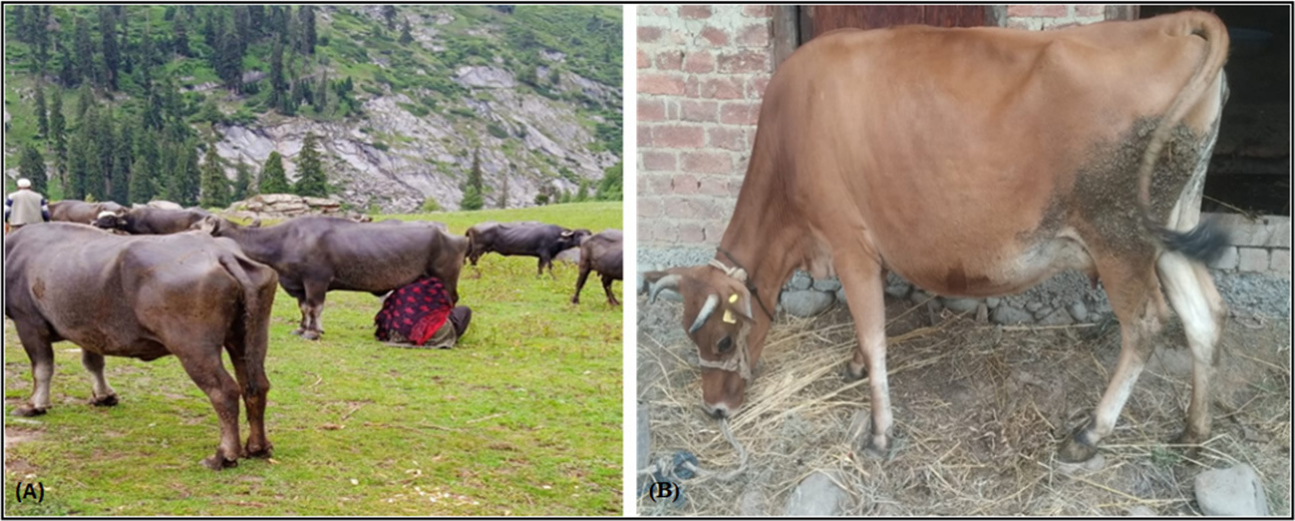
**(A)** The Bakarwal lady hand milking from buffalo in Jammu region **(B)** cow with root of *Ulmus wallichiana* in neck to protect from black magic and evil eye in Kashmir region.

During our research, we discovered certain species that had never been reported for ethno-veterinary practices in these biogeographic regions. The seeds of *Amaranthus blitum*, the leaves of *Morus alba, Ficus palmata, Vitex negundo, Juniperus semiglobosa*, the roots of *Ulmus wallichiana*, and *Rumex nepalensis* were used against black magic and evil eyes. The mixture of burning powder is then burnt to produce smoke in traditional fire pots called *kangri* and circulated seven times around the animal, particularly cows, to get rid of black magic and the evil eye (Figure 7). *Heracleum candicans* were used to increase the milk production of cattle. The sap of leaves and stems from *Sambucus wightiana* was applied for wound healing in cattle. The unique fodder preparation from the seeds of *Brassica campestris*, locally called *Kaj*, is traditionally used as a nutritive food for cattle in the winter season. The various dry fodder preparations (*gaaslov, pan baath, kaandbaath, Lovgooad, Karb*, and *Phungma*) are here reported for the first time from the Himalayan region. *Gasslov* is made by bundling six to ten handfuls of *Oryza sativa* and drying it in the sun; *pan baath* is obtained by gathering the leaves of *Salix alba* along with small branches and dried, used in winters, especially for sheep and goats; *lovgooad* is made by drying and bundling different species such as *Trifolium fragiferum, Trifolium pretense, Trifolium repens*, and *Cynodon dactylon*.

### Ethnopharmacological relevance

One purpose of ethnopharmacology is to expand our understanding of the pharmacology of traditional medicinal plant use, particularly for the benefit of marginalized and impoverished rural populations. We reported 22 diseases (gastrointestinal issues, wounds, weakness, urine issues, swelling, skin diseases, sexual issues, gynecological issues, foot and mouth diseases, endoparasites, nasal worms, kidney stones, fever, cough, constipation, black quarter, red-water disease, tongue infections, fractures, liver issues, eye issues, and joint issues) (Supplementary Table 1) as treated with the documented species. Alawa et al. (56) reported that 21 aliments of animals were cured by a traditional medical practice in Nigeria. Among the documented diseases, gastrointestinal issues were found to be treated with the maximum number of species (*n* = 12), followed by urinary issues treated by species (*n* = 11). Endoparasites were second in importance, treated with eight species. Cough was treated using six plant species, which included *Berberis lycium, Vitex negundo, Nasturtium officinale, Bauhinia variegata, Cuscuta capitata*, and *Justicia adhatoda*. Gastrointestinal problems were common diseases also reported by Aziz et al. (18), who also found most medicinal plants (32) are used for gastric problems in the Pakistan Himalayas. Similarly, Suroowan et al. (57) reported that gastric diseases were commonly treated by medicinal plants in South Asia. It is important to mention that scientific phytochemical evaluation of these species is essential, as this might provide novel compounds with potential medicinal attribution, which, in turn, can prove beneficial for going against antibiotic-resistant pathogens, which are of prime concern to the medical world.

### Plant resource usage across the region

**Figure 8:**
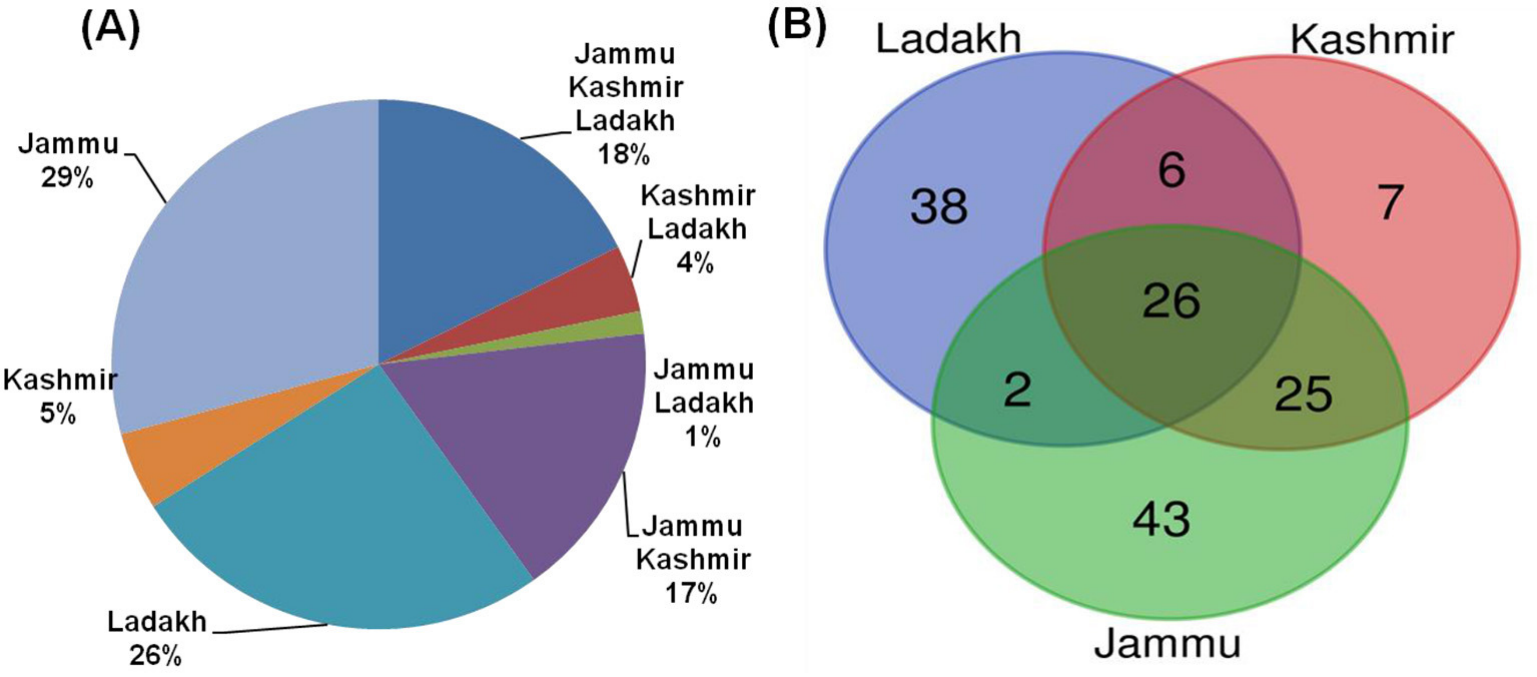
**(A)** Showing percentage of similarity **(B)** Venn diagram showing pattern of ethno-veterinary usage pattern of plant resources in different regions.

The Jammu and Kashmir region showed greater similarity (17% of joint species), whereas the least similarity (1%) was observed between the Jammu and Ladakh regions (Figure 8A). The Venn diagram (Figure 8B) shows that the maximum number of species (26%, 38 Spp.) was uniquely used in the Ladakh region, while the Kashmir region reported a minimum of (5%, 7 Spp.) of species uniquely used by local residents. A cross-cultural assessment of plant resources revealed that 26 plants (18%) overlapped between the three regions (Figures 8A,B). The reason for both Kashmir and Jammu sharing their culture lies in frequent intermarriage and the similarity in religions, ethnic groups, and easy accessibility, whereas Jammu and Ladakh had distinct cultural identities and hence showed the least relationship. The common species were especially used for fodder and magical uses. Aziz et al. (18) and Aziz et al. (58) conducted a similar cross-cultural analysis in the Pakistan Himalaya and concluded that this trend indicates that location and ecology, as well as ethnicity and cultural practices, have shaped traditional veterinary herbal knowledge among the local inhabitants for a long time. Abidin et al. (59) from Southwest Pakistan and Kunwar et al. (60) from Nepal Himalaya revealed similar findings, which confirm our results from the Himalayan region of Jammu and Kashmir.

### A quantitative ethnoveterinary approach

**Figure 9:**
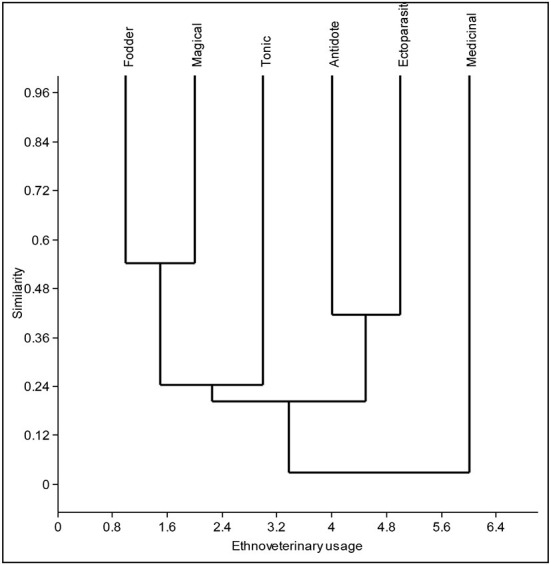
Cluster diagram of the different provisioning services based on plant usage pattern in different regions.

**Figure 10:**
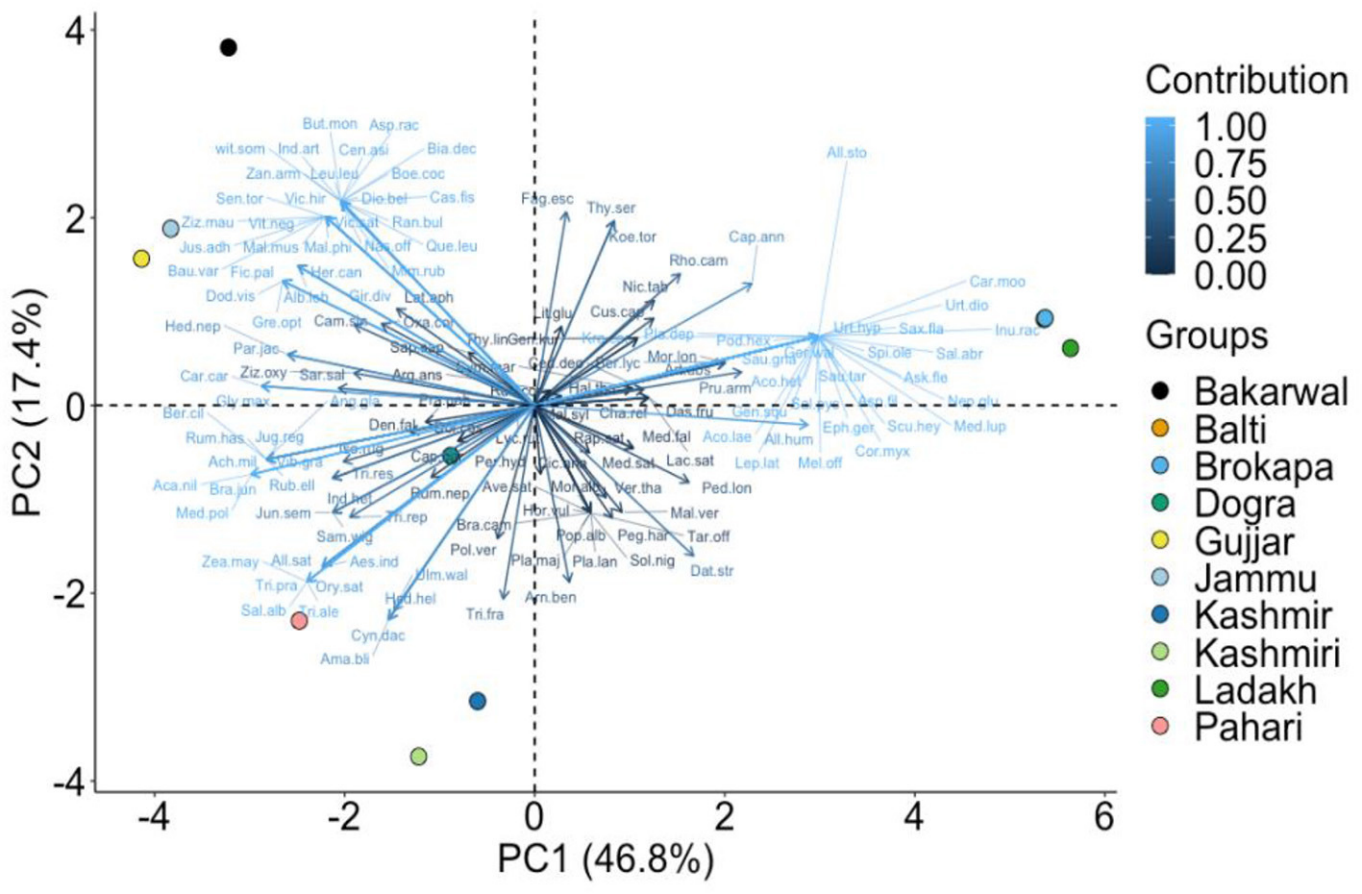
PCA diagram representing clustering of plant species among 3 regions and ethical groups. The complete name of species is show in Supplementary Table 1.

**Figure 11:**
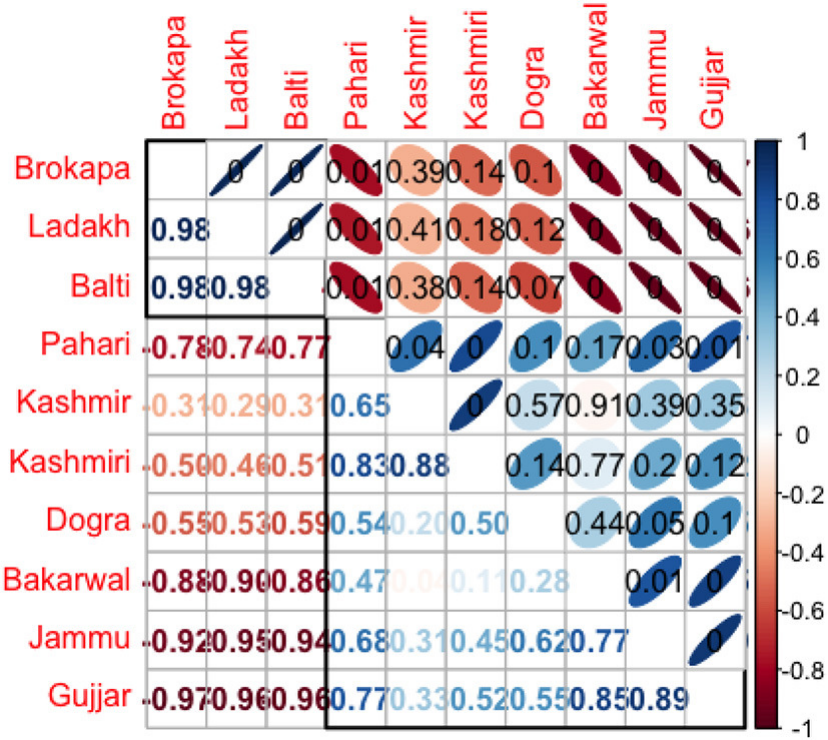
Correlogram showing the Pearson correlation results between ethical groups evaluated in the 3 regions.

The cluster analyses resulted in four major clusters of six ethno-veterinary uses based on floristic similarity (Figure 9). The Sorensen's similarity index recognized four clusters, i.e., cluster one – fodder and magical, cluster two – tonic, used to increase strength in animals, cluster three – antidote and ectoparasite, and the fourth cluster included species used for medicinal purposes (Supplementary Table 1 and Figure 9). We summarized the utilization of plant resources based on preference, seasonality, culture, availability in three regions, along with a comparative analysis of ethnic groups. The PCA showed considerable variation in different regions and ethnic groups, and specific groups of species were more closely related to a particular region and ethnic group than others (Figure 10). For example, PC1 (46.8%) and PC2 (17.4%) of species distribution in the biplot, in which Jammu and Kashmir region species grouped on one side of the PCA and Ladakh formed a separate cluster based on species presence/absence (Figure 10). The usage of plant resources in similar regions across ethnic groups varied. The Gujjar, Bakharwal, Pahari, Brokpa, and Balti had more traditional ethno-veterinary knowledge as compared to the Kashmiri and Dogra. This can be explained by the fact that Gujjar, Bakharwal, Pahari, Brokpa, and Balti are mostly found in remote mountainous areas (Table 1) that lack basic modern facilities and thus rely heavily on natural resources, in contrast to the Kashmiri and Dogra. This was further supported by the Pearson correlation coefficient, describing the strength and direction of an association between ethnic groups and regions (Figure 11). The *P*-values are displayed at the top, and Pearson correlation coefficients are displayed at the bottom (Figure 11). Various ethno-veterinary ethnoveterinary studies, such as Majekodunmi et al. (61) from West Africa, Catley (62) from East Africa, Raza et al. (63) from Pakistan's Cholistan desert, Rezende et al. (64) from Minas Gerais, and Haq et al. (65, 66) from the Himalayas, have shown similar hierarchical clustering, multidimensional scaling, and associations.

### Plant resources as a source for local livelihood

Plant resources were not only a source of fodder and medicine but also an opportunity for income generation. The straw of plants like *Oryza sativa, Zea mays, Hordeum vulgare, Avena sativa*, and the seed residue from *Brassica campestris—*locally called *Kaj—*were commercialized for income generation. In addition, various species of *Trifolium*, along with *Cynodon dactylon, Medicago falcata*, and *Melilotus officinalis*, formed a unique fodder preparation called *lov, karbbugsukrtsua*, which is also used as a source of income. Similarly, Reynolds et al. (67) and Balehegn et al. (68) reported that foraging enhanced livelihoods in low- and middle-income nations. Even the dry twigs with leaves of tree species such as *Salix alba* and *Morus alba* were used as fodder and a source of income, as they were considered the most desirable and cheapest sources of sustenance for animals in the winter season. A steady supply of high-quality fodder in appropriate amounts is critical in cattle rearing. The locals favored wild plants (*Heracleum candicans* and *Taraxacum officinale*) and believed that milk production could be increased by using wild, high-quality nutritional plants, thus increasing their source of income. Similar to Franzel et al. (69), fodder species improved livelihood conditions in Africa by boosting milk output.

## Conclusion

The indigenous communities of Jammu and Kashmir's Himalayas are dependent on plant resources for ethno-veterinary practices and income creation. In comparison to the Kashmiri and Dogra ethnic groups, the knowledge of traditional ethno-veterinary practices was higher in the Gujjar, Pahari, Bakharwal, Brokpa, and Balti ethnic communities. Our research aims to help prevent traditional ethno-veterinary knowledge from going extinct. A number of plants were employed in magical systems, with some important plants being used as galactagogues in cattle. To ensure the safe and continuous secure use of the documented ethno-veterinary practices, critical toxicological investigations would be of interest. Aside from scientific substantiation for the use of these plants, research into constituents in specific regions, including changes in harvesting times and clinical efficacy, should also be investigated. It is critical to educate the rural population about the importance of traditional ethno-veterinary knowledge and motivate them to conserve the natural flora to share and preserve this knowledge. Traditional knowledge documentation serves a range of functions, including preserving it for future generations, securing it by putting it in the public domain, and using it as a starting point for additional research and conservation measures.

## Data availability statement

The original contributions presented in the study are included in the article/Supplementary material, further inquiries can be directed to the corresponding author/s.

## Ethics statement

Ethical review and approval was not required for the animal study because this is a survey based study and dose not require approval from Ethical Committee. Written informed consent was obtained from the owners for the participation of their animals in this study.

## Author contributions

Study design and conceptualization: SH, RA, AM-d, MA, and JP. Data collection: SH, MH, and UY. Analysis: SH, EC, JL, MA, and JP. Visualization: SH, MH, MW, UY, EC, and RA. Initial draft and sources: SH. Supervision and funding acquisition: MA and AM-d. Proofreading and validation: RA, EC, MM, RB, MA, JL, JP, and AM-d. All authors read and approved the final manuscript.

## Funding

This research was funded by project IGYMERA, under grant agreement 101030604. AM-d is a recipient of a postdoctoral Marie Curie fellowship under grant agreement 101030604 (IGYMERA).

## Conflict of interest

Author MH and SH was employed by Clybay Research Private Limited. The remaining authors declare that the research was conducted in the absence of any commercial or financial relationships that could be construed as a potential conflict of interest.

## Publisher's note

All claims expressed in this article are solely those of the authors and do not necessarily represent those of their affiliated organizations, or those of the publisher, the editors and the reviewers. Any product that may be evaluated in this article, or claim that may be made by its manufacturer, is not guaranteed or endorsed by the publisher.
