# Supplementary material for: Quantitative ethnoveterinary study on plant resource utilization by indigenous communities in high-altitude regions
Source: Front Vet Sci. 2022 Oct 6;9:944046. doi: 10.3389/fvets.2022.944046 (PMC9583879; doi:10.3389/fvets.2022.944046)
Supplement: Supplementary file 1 [file Table_1.docx]

**Table 2**. List of plants including their botanical names, voucher numbers, family names, bio-geographic regions, vernacular names, and part used, preparation, ethno-usage as well as medical purposes in different ethical groups of J&KH.

| **Name of species**  **(Family)**  **Voucher No.** | **Bio-geographic region** | | | **Local name** | **Part used** | **Preparation**  **embodiment** | **Ethno-usage** | **Ethno-pharmacological usage** | **Ethnic groups** |
| --- | --- | --- | --- | --- | --- | --- | --- | --- | --- |
|  | **Jam** | **Kas** | **Lad** |  |  |  |  |  |  |
| *Acacia nilotica* (L.) Willd. ex Delile  (Fabaceae)  SMH-701 | Y | Y | N | Kikar (K)  Keekar (J) | Leaves | Raw | Raw fresh leaves are given as fodder and to enhance milk yield. | -------------------- | Bakarwal  Kashmiri  Dogra  Pahari |
| *Achillea millefolium* L.  (Asteraceae)  SMH-702 | Y | Y | N | Chuang (J)  Bair guer (K) | Leaves | Powder | Leaves are given as fodder. | Leaves are sun dried, powdered and dissolved in water and given three times a day to treat spasmodic pain. | Bakarwal  Kashmiri |
| *Aconitum heterophylloides* (Brühl) Stapf  (Ranunculaceae)  SMH-703 | N | N | Y | Bish (L) | Root  Leaves | Paste | Leaves are given as fodder. | Root are made into paste and applied topically to get rid from maggots. | Brokapa  Balti |
| *Aconitum laeve* Royle  (Ranunculaceae)  SMH-704 | N | N | Y | Zoaneer (L) | Leaves | Raw | Leaves are given raw as fodder. | -------------------- | Brokapa  Balti |
| *Aesculus indica* (Wall. ex Cambess.) Hook  .  (Sapindaceae)  SMH-110 | Y | Y | N | Handoon (J)  Gurpan (K) | Seeds | Powder | -------------------- | Seeds grinded to powder and give with Luke warm water to treat infertility and to maintain body heat. | Kashmiri  Pahari |
| *Albizia lebbeck* (L.) Benth.  (Fabaceae)  SMH-705 | Y | N | N | Sariin (J) | Leaves | Raw | Raw leaves are given as fodder. | -------------------- | Bakarwal  Pahari |
| *Allardia stoliczkae* C.B.Clarke  (Asteraceae)  SMH-706 | N | N | Y | Solo-marpo (L) | Shoot | Decoction | -------------------- | Decoction obtained from shoots is given twice a day to treat pain due to wound. | Brokapa  Balti |
| *Allium humile* Kunth  (Amaryllidaceae)  SMH-164 | N | Y | Y | Skotse (L)  Jangli praan (K) | Bulb | Decoction | --------------------. | Decoction obtained from dried bulb is given after delivery to regain strength. | Kashmiri  Pahari  Brokapa  Balti |
| *Allium sativum* L.  (Amaryllidaceae)  SMH-707 | Y | Y | N | Rhun (K)  Lsan (J) | Bulb | Paste | -------------------- | Blub is made into paste, mixed with oil and applied over body to get rid from ticks. | Kashmiri  Pahari |
| *Amaranthus blitum* L.  (Amaryllidaceae)  SMH-708 | N | Y | N | Kanhaar/ Ganhar (K) | Seeds | Raw | Seeds are burned to produce smoke which is circulated seven times around cattle to wind off the ill effects of black magic. | -------------------- | Kashmiri  Pahari |
| *Angelica glauca* Edgew.  (Apiaceae)  SMH-203 | Y | Y | N | Sapsade (J)  Chorak (K) | Rhizome | Powder | -------------------- | Powder of rhizome is added with feed to overcome weakness. | Bakarwal  Kashmiri  Pahari |
| *Argentina anserina* (L.) Rydb.  (Rosaceae)  SMH-709 | Y | Y | Y | Penma (J)  Troma (L)  Sapaid pan (K) | Leaves | Decoction | -------------------- | Leaves are made into decoction and given twice a day for diarrhoea. | Bakarwal  Kashmiri  Pahari  Brokapa  Balti |
| *Arnebia benthamii* (Wall. ex G.Don) I.M.Johnst.  (Boraginaceae)  SMH-710 | Y | Y | Y | Kahzaban (K)  Ratanjot (J)  Ulte bhutke (L) | Leaves | Herbal tea | Leaves boiled for half an hour and then given in cold. | -------------------- | Kashmiri  Dogra  Pahari  Brokapa |
| *Artemisia absinthium* L  (Asteraceae)  SMH-125 | N | N | Y | Tartiha (L) | Leaves | Extract | -------------------- | Leaves are grinded into paste squeezed and the obtained sap is applied over the affected part to treat mouth disease. | Balti |
| *Askellia flexuosa* (Ledeb.) W.A.Weber  (Asteraceae)  SMH-711 | N | N | Y | Omsa (L) | Leaves | Raw | Raw leaves are given as fodder. | -------------------- | Brokapa  Balti |
| *Asparagus filicinus* Buch.-Ham. ex D.Don  (Asparagaceae)  SMH-712 | N | N | Y | Phelgasse (L) | Whole plant | Paste | -------------------- | Fresh plants are made into paste and given to animal with weak uterus. | Brokapa  Balti |
| *Asparagus racemosus* Willd.  (Asparagaceae) | Y | N | N | Sainspii (L) | Root | Decoction | Decoction obtained from the roots is given orally to increase milk production. | Decoction obtained from the roots is given orally to treat liver problems. | Bakarwal |
| *Avena sativa* L.  (Poaceae)  SMH-713 | Y | Y | Y | Jayee (J)  Yukpo (L)  Oats (K)  Janni (K) | Whole plant | Raw | Straw is stored as fodder for winter and is called as *Yukpo Rtsua* | -------------------- | Kashmiri  Dogra  Pahari  Brokapa  Balti |
| *Bauhinia variegata* L.  (Fabaceae) | Y | N | N | Kalari (J) | Leaves  Flower  Buds | Raw | Leaves are given as fodder. | Fresh flowers and buds are given to treat Cough and purify blood. | Bakarwal  Dogra |
| *Berberis lycium* L.  (Berberidaceae)  SMH-306 | N | N | Y | Skerpa (L) | Bark  Stem | Raw | -------------------- | Bark and stem are dried and given as raw to treat factures, cough and sprains. | Balti |
| *Bergenia ciliata* (Haw.) Sternb.  (Saxifragaceae)  SMH-202 | Y | Y | N | Sapdotary (J)  Zakhm-e- hayat (K) | Root | Raw | -------------------- | Pieces of fresh roots are mixed with flour of *Hordeurn vulgare* and *Zea mays* to treat weakness. | Bakarwal  Kashmiri  Pahari |
| *Biancaea decapetala* (Roth) O.Deg.  (Fabaceae)  SMH-714 | Y | N | N | Arlu (J) | Whole plant | Raw | whole plant used as fodder | -------------------- | Bakarwal |
| *Boerhavia coccinea* Mill.  (Nyctaginaceae)  SMH-715 | Y | N | N | Itsit (J) | Root | Crushed | -------------------- | Crushed roots are wrapped in wheat bread and given to cows or buffaloes to treat Black quarter disease. | Bakarwal |
| *Brassica campestris* L.  (Brassicaceae)  SMH-523 | Y | Y | Y | Telgul (K)  Sarsoon (J)  Rutagaba (L) | Seeds | Paste | Seeds are extracted for oil and the left residue is used for feed in Kashmir locally called *KHAJ* which is used as fodder and keeps body warm during winter. | Seeds roasted grinded into fine powder added with some water to make paste which is used to treat diarrhoea. | Kashmiri  Dogra  Pahari  Brokapa  Balti |
| *Brassica juncea* (L.) Czern.  (Brassicaceae)  SMH-716 | Y | Y | N | Sarsoon (J)  Telgul (K) | Seeds | Oil | -------------------- | Extracted oil is mixed with mustard oil and applied topically to get against ectoparasites. | Bakarwal  Kashmiri  Dogra  Pahari |
| *Butea monosperma* (Lam.) Kuntze  (Fabaceae)  SMH-717 | Y | N | N | Palas (J) | Seed | Crushed | -------------------- | Crushed seeds orally used to treat skin diseases. | Bakarwal |
| *Camellia sinensis* (L.) Kuntze  (Theaceae)  SMH-718 | Y | N | N | Chai kul  Chaa (J) | Leaves | Tea | -------------------- | Leaves boiled for an hour and then given to animal for the treatment of urine blockage. | Bakarwal  Dogra  Pahari |
| *Capsella bursa-pastoris* (L.) Medik.  (Brassicaceae)  SMH-427 | Y | Y | N | Kralmond (K)  Chambraka (J) | Leaves  Shoots | Raw | Leaves and shoots are given as fodder and to increase milk production. | -------------------- | Bakarwal  Kashmiri  Pahari  Dogra |
| *Capsicum annuum* L.  (Solanaceae)  SMH-719 | Y | N | Y | Merchwangun (K)  Merchi (K)  Merch (K)  Ohm Jolika (L) | Fruit | Raw | -------------------- | Red chilli and salt is fed to animal early morning empty stomach to get against endoparasites and liver flukes. | Pahari  Dogra  Brokapa  Balti |
| *Carex moorcroftii* Falc. ex Boott  (Cyperaceae)  SMH-720 | N | N | Y | Long- ma (L) | Whole plant | Raw | Raw whole plant is used as fodder. | -------------------- | Brokapa  Balti |
| *Carum carvi* L.  (Apiaceae)  SMH-104 | Y | N | N | Konagy (J) | Seeds | Crushed | -------------------- | Seeds are crushed and given in winter to treat weakness. | Bakarwal  Kashmiri  Dogra  Pahari |
| *Cassia fistula* L.  (Fabaceae)  SMH-311 | Y | N | N | Karangal (J) | Leaves  Flower | Raw  Paste | Raw leaves are given as fodder. | Paste obtained from the flower is applied topically to treat headache. | Bakarwal |
| *Cedrus deodara* (Roxb. ex D.Don) G.Don  (Pinaceae)  SMH-721 | Y | Y | N | Sangul (K)  Kelam (J) | Leaves  Seeds | Raw | Raw leaves are given as fodder. | Seeds are extracted, to obtained oil which is applied topically to treat skin diseases. | Bakarwal  Pahari |
| *Centella asiatica* (L.) Urb.  (Apiaceae)  SMH-722 | Y | N | N | Brahmi booti (J) | Leaves | Powder | -------------------- | Dried leaves are powdered to treat wounds. | Bakarwal |
| *Chaerophyllum reflexum* Lindl.  (Apiaceae)  SMH-723 | Y | Y | Y | Chervil (K)  Chervili (J)  Lcha-wa or Young (L) | Roots | Powder | -------------------- | Roots are crushed and powdered and given twice a day for a week to treat urine blockage. | Bakarwal  Pahari  Balti |
| *Cicer anatolicum* Alef.  (Fabaceae)  SMH-724 | N | N | Y | Sari | Leaves | Raw | Raw leaves are given as fodder. | -------------------- | Balti  Brokapa |
| *Cordia myxa* L.  (Boraginaceae) | N | N | Y | Safestan  Lasura (L) | Seeds | Poultice | -------------------- | Leaves are made into poultice and then applied topically to treat swelling. | Brokapa  Balti |
| *Cuscuta capitata* Roxb.  (Convolvulaceae)  SMH-725 | N | N | Y | Deelasazin  Amarlata (L) | Whole plant | Powder | -------------------- | Whole plant is powdered and given with water to treat Cough and fever. | Brokapa |
| *Cymbopogon martinii* (Roxb.) Wats  (Poaceae)  SMH-726 | N | N | Y | Lchiesayang (L) | Leaves | Raw | Long leaf is tied around the horn of cattle to overcome evil eye. | -------------------- | Balti  Brokapa |
| *Cynodon dactylon* (L.) Pers.  (Poaceae)  SMH-727 | N | Y | N | Habil kanji (K) | Whole plant | Raw | Plant is dried and used as fodder. It is a constituent species of unique fodder preparation locally called as *LOV.* | -------------------- | Kashmiri  Pahari |
| *Dasiphora fruticosa* (L.) Rydb.  (Rosaceae)  SMH-728 | Y | Y | Y | Chai kul (K)  Panzpatar (L)  Rattenjot (J) | Rhizome | Decoction | -------------------- | Rhizome is boiled in water and given to animal to treat indigestion. | Bakarwal  Kashmiri  Pahari  Brokapa |
| *Datura stramonium* L.  (Solanaceae)  SMH-205 | N | Y | Y | Esman (J)  Dutur (K) | Seed | Raw | The seeds are burned to produce smoke, which is circulated seven times around the cattle to wind off evil. | -------------------- | Kashmiri  Balti |
| *Dendrophthoe falcata* (L.f.) Ettingsh.  (Loranthaceae)  SMH-729 | Y | N | N | Banda  Hawaipatta (J) | Whole plant | Raw | -------------------- | Raw plant is given to animals for the retention of placenta. | Dogra  Pahari |
| *Dioscorea belophylla* (Prain) Voigt ex Haines  (Dioscoreaceae)  SMH-730 | Y | N | N | Tarad (J) | Tuber | Raw | -------------------- | Raw tubers are given orally to treat dysentery and Heart troubles. | Bakarwal |
| *Dodonaea viscosa* Jacq.  (Sapindaceae)  SMH-731 | Y | N | N | Saentha  Manjha (J) | Leaves | Powder | -------------------- | Leaves are sun dried, powdered, mixed with luke warm water and given twice a day to get against intestinal worms. | Bakarwal  Dogra  Pahari |
| *Dolomiaea costus* (Falc.) Kasana & A.K.Pandey  (Asteraceae)  SMH-732 | Y | Y | N | Koth (K)  Kuste Himi (J) | Stem  Roots | Powder  Paste | -------------------- | Rhizome is grinded into fine powder and is given with water to treat coldness.  Paste of root of is applied topically to get against maggots. | Bakarwal  Kashmiri  Pahari  Dogra |
| *Ephedra gerardiana* Wall. ex Klotzsch & Garcke  (Ephedraceae)  SMH-733 | N | N | Y | Sephat, Tse-phat (L) | Aerial Part | Raw  Decoction | Raw aerial part is given as fodder. | Decoction obtained from the aerial part is used against lung problems | Balti  Brokapa |
| *Fagopyrum esculentum* Moench  (Polygonaceae)  SMH-449 | Y | N | Y | Chok drou (J)  Trumb (J)  Phaphra (L)  Bro, Brozun (L) | Leaves | Raw | Raw leaves are given as fodder. | Raw leaves are fed to animal twice daily for 5 days to treat foot and mouth disease (FMD). | Bakarwal  Dogra  Pahari  Brokapa  Balti |
| *Ficus palmata* Forssk  (Moraceae)  SMH-319 | Y | N | N | Anjeer (J) | Leave | Raw | Fresh leaves are enchanted and given orally to get against evil eye, also given as such as fodder. | -------------------- | Bakarwal  Dogra  Pahari |
| *Gentiana kurroo* Royle  (Gentianaceae)  SMH-734 | N | Y | Y | Butein (L)  Neel kanth (J)  Tikta (J) | Whole plant | Raw | -------------------- | Whole plant is given to animal 2 to 3 times a day to treat liver fluke problem. | Bakarwal  Pahari  Brokapa  Balti |
| *Gentiana squarrosa* Ledeb.  (Gentianaceae)  SMH-735 | N | N | Y | Ziang (L) | Roots | Powder | -------------------- | Shade dried roots are powdered and given with feed to calm the cattle during fracture. | Brokapa  Balti |
| *Geranium wallichianum* D.Don ex Sweet  (Geraniaceae)  SMH-736 | N | N | Y | Ratan jote (L) | rhizome | Powder | -------------------- | Rhizome is grinded into fine powder and is given with water to treat weakness. | Brokapa  Balti |
| *Girardinia diversifolia* (Link) Friis  (Urticaceae)  SMH-737 | Y | N | N | Bichu  Cell kinji (J) | Root | Powder | -------------------- | Crushed, powdered roots are made into small balls and given twice 3 balls for 3 days to treat red water disease. | Bakarwal  Pahari |
| *Glycine max* (L.) Merr.  (Fabaceae)  SMH-738 | Y | Y | Y | Bhetmas (J)  Muth (K)  Frijol de so (L) | Seeds | Aquafaba | -------------------- | Seeds are kept in water overnight and then given to the animal to treat post-delivery complications. | Bakarwal  Kashmiri  Dogra  Pahari  Brokapa |
| *Grewia optiva* J.R.Drumm. ex Burret  (Malvaceae)  SMH-739 | Y | N | N | Dahaman (J) | Leaves | Raw | Raw leaves are made into amulet to treat evil eye. Raw leaves are also given as fodder. | -------------------- | Bakarwal  Dogra  Pahari |
| *Haloxylon thomsonii* Bunge ex Boiss.  (Amaranthaceae)  SMH-740 | N | N | Y | Jema (L) | Whole plant | Raw | Raw whole plant is used as fodder. | -------------------- | Brokapa  Balti |
| *Hedera helix* L.  (Araliaceae)  SMH-741 | Y | Y | N | Bail (J)  Banbatkari (J)  Band (K) | Leaves | Paste | -------------------- | Leaves are made into paste and applied on the area of snake bite. | Kashmiri  Pahari |
| *Hedera nepalensis* K.Koch  (Araliaceae)  SMH-742 | Y | Y | N | Karuri (J)  Ktambari (J, K) | Leaves | Paste | -------------------- | Leaves are made into paste and applied on the area of bite. | Bakarwal  Pahari |
| *Heracleum candicans* Wall. ex DC.  (Apiaceae)  SMH-454 | Y | N | N | Wanntamokh (J) | Leaves | Raw | Leaves are given to increase milk production. | -------------------- | Bakarwal  Pahari |
| *Hordeum vulgare* L  (Poaceae)  SMH-743 | Y | Y | Y | Yangma (L)  Nas (J,K)  Jaoon (J,K) | Whole plant | Raw | Straw is stored for winter and is called as *Phoong-Ma* in Ladakh. | -------------------- | Kashmiri  Dogra  Pahari  Brokapa  Balti |
| *Indigofera articulata* Gouan  (Fabaceae)  SMH-744 | Y | N | N | Neel (J) | Leaves | Raw | Leaves used as fodder | -------------------- | Bakarwal |
| *Indigofera heterantha* L.  (Fabaceae)  SMH-745 | Y | Y | Y | Kawpumble (K)  Keynthi (J)  Kanhchu (L) | Roots  Leaves | Raw  Half-cooked | Raw leaves are given as fodder. | Mashed half-cooked roots are used to treat constipation. | Kashmiri  Pahari  Balti  Brokapa |
| *Inula racemosa* Hook.f.  (Asteraceae)  SMH-176 | N | N | Y | Maleen (L) | Rhizome | Powder | -------------------- | Powder of rhizome is given with water to treat liver fluke issues. | Brokapa  Balti |
| *Isodon rugosus* (Wall. ex Benth.) Codd  (Lamiaceae)  SMH-746 | Y | Y | N | Suli (K)  Sloi (K)  Maldah (J)  Sheshak (J) | Leaves | Raw | -------------------- | Raw leaves are fed twice daily for 3 days to treat endo-parasites. | Pahari |
| *Juglans regia* L.  (Juglandaceae)  SMH-326 | Y | Y | N | Doon (K)  Khooad (J,K)  Akhroot (K, J) | Fruit exocarp | Raw | -------------------- | Raw exocarp of raw nut is used treat the pus in toes. | Bakarwal  Kashmiri  Pahari |
| *Justicia adhatoda* L.  (Acanthaceae)  SMH-747 | Y | N | N | Bekarh (J) | Leaves | Paste | -------------------- | Paste obtained from fresh leaves are used to treat wounds. | Bakarwal  Dogra |
| *Juniperus semiglobosa* Regel  (Cupressaceae)  SMH-748 | N | Y | N | Chillee (K) | Leaves  Twigs | Raw | Raw dried leaves and twigs are burn to produce smoke and burn in traditional fire pot called *Yazbandaain* to treat evil eye disease. | -------------------- | Bakarwal  Kashmiri  Pahari |
| *Koenigia tortuosa* (D.Don) T.M.Schust. & Reveal  (Polygonaceae)  SMH-749 | Y | Y | Y | Serpalulu (L) Itimeng (L)  Palkach (J)  Chikro (K) | Whole plant | Decoction | -------------------- | Whole plant is made into decoction which is used to treat painful urination. | Bakarwal  Brokapa  Balti |
| *Krascheninnikovia ceratoides* (L.)  (Amaranthaceae)  SMH-750 | N | N | Y | Gapson  Gabsan (L) | Leaves | Raw | Raw leaves are used as fodder, also given to increase milk yield in goats. | -------------------- | Brokapa  Balti |
| *Lactuca sativa* L.  (Asteraceae)  SMH-751 | Y | Y | Y | Bandgopi (K)  Dums (L)  Salad (J) | Leaves | Raw  Decoction | Raw leaves are given as fodder. | Decoction is given twice a day for three consecutive days to treat fever. | Kashmiri  Pahari  Brokapa |
| *Lathyrus aphaca* L.  (Fabaceae)  SMH-752 | Y | N | N | Jungli matar (J) | leaves | Raw | Raw leaves are given as fodder. | -------------------- | Bakarwal  Pahari  Dogra |
| *Lepidium latifolium* L.  (Brassicaceae)  SMH-753 | N | N | Y | Seoji  Jungli haloon  Seoji  Sangso  Sauson (L) | Whole plant | Paste | -------------------- | Whole plant is made into paste which is applied to the fractured body part and to the joints. | Brokapa  Balti |
| *Leucaena leucocephala* (Lam.) de Wit  (Fabaceae)  SMH-754 | Y | N | N | Pallai (J) | Stem  Leaves | Raw | Raw leaves and stem are used as fodder. | -------------------- | Bakarwal |
| *Litsea glutinosa* (Lour.) C.B. Rob.  (Lauraceae)  SMH-755 | N | N | Y | Rahen  Medasak (L) | Root  Bark | Powder | -------------------- | After making a proper adjustment of the dislocated joints, the powdered bark or root is given orally for three days. | Brokapa |
| *Lycium ruthenicum* Murray  (Solanaceae)  SMH-756 | N | Y | Y | Umila (J)  Hei guo (L) | Leaves | Decoction | -------------------- | Decoction obtained from leaves is given twice a day to treat urine blockage. | Pahari  Brokapa |
| *Malaxis muscifera* (Lindl.) Kuntze  (Orchidaceae)  SMH-757 | Y | N | N | Jeewak (J) | Roots | Decoction | -------------------- | Decoction is given twice a day to treat constipation. | Bakarwal  Dogra |
| *Mallotus philippensis* (Lam.) Müll.-Arg.  (Euphorbiaceae)  SMH-758 | Y | N | N | Kameela (J) | Fruit | Powder | -------------------- | Fruits are shade dried, powder given orally with water to expel worms. | Bakarwal  Dogra |
| *Malva sylvestris* L.  (Malvaceae)  SMH-759 | N | Y | N | Sotsal  Gur sachal(K) | Leaves | Paste | Paste of young grinded leaves given to calf for body strength. | -------------------- | Bakarwal  Kashmiri |
| *Malva verticillata* L.  (Malvaceae)  SMH-329 | N | Y | Y | Cam-pa-ma-ning (L)  Sochal (J) | Whole plant | Decoction | -------------------- | Decoction is given twice a day to treat stomach disorders. | Kashmiri  Balti |
| *Medicago falcata* L.  (Fabaceae)  SMH-760 | Y | Y | Y | Ole,Buksug (L)  Saridha (J)  Tailhol (K) | Whole plant | Raw | Straw is stored as fodder for winter and is called as *Mal-Buksug* in Ladakh. | -------------------- | Kashmiri  Dogra  Brokapa  Balti |
| *Medicago lupulina* L.  (Fabaceae)  SMH-761 | N | N | Y | Burahang (L) | Whole plant | Raw | Raw whole plant is used as fodder. | -------------------- | Brokapa  Balti |
| *Medicago polymorpha* L.  (Fabaceae)  SMH-762 | Y | Y | N | Saridha (J)  Trehul (K) | Whole plant | Raw | Raw whole plant is used as fodder. | -------------------- | Bakarwal  Kashmiri  Dogra  Pahari |
| *Medicago sativa* L  (Fabaceae)  SMH-763 | Y | Y | Y | Chunpo (J)  Saridha (K)  Oal (L) | Stem | Raw | Raw stem is used as fodder. It is also a constituent species of unique preparation called *SROLO (RZASOT*) in Ladakh. | -------------------- | Kashmiri  Dogra  Brokapa  Balti |
| *Melilotus officinalis* (L.) Lam.  (Fabaceae)  SMH-764 | N | N | Y | Bugsuk  Skambo | Aerial part | Raw | Raw aerial parts are dried and commonly used as fodder in winter season. It is also a constituent species of the unique feed preparation called *SROLO-RZASO* in Ladakh. | -------------------- | Brokapa  Balti |
| *Mimosa rubicaulis* Lam.  (Fabaceae)  SMH-765 | Y | N | N | Raal (J) | Bark | Powder | -------------------- | Powder obtained from the bark is given orally to treat inflammation. | Bakarwal |
| *Morina longifolia* Wall. ex DC.  (Caprifoliaceae)  SMH-191 | N | N | Y | Agzaima (L) | Seeds | Oil | -------------------- | Oil is obtained by crushing the seed is applied on skin to overcome lice problems. | Brokapa |
| *Morus alba* L.  (Moraceae)  SMH-334 | Y | Y | Y | Tulpa (L)  Shahtoot (J, K)  Shahtul (K) | Leaves  Root | Extract  Raw | Raw roots are used in the form of amulet to overcome black magic. Leaves of *Morus alba* are the constituent ingredients of unique feed preparation called *SROLO-RZASOT* in Ladakh. | Fresh leaves are grinded squeezed and then the obtained sap is applied to feet injuries. | Kashmiri  Dogra  Pahari  Brokapa  Balti |
| *Nasturtium officinale* W.T. Aiton  (Brassicaceae)  SMH-766 | Y | N | N | Choo (J) | Leaves | Raw | Raw leaves used as fodder. | Raw leave are used to treat cough, cold and blood purify. | Bakarwal |
| *Nepeta glutinosa* Benth.  (Lamiaceae)  SMH-767 | N | N | Y | Sankukorum  Gimanka  Jatukpa (L) | Leaves  Young twigs | Raw | Raw leaves and young twigs are used as fodder and used to increase strength in goats. | -------------------- | Brokapa  Balti |
| *Nicotiana tabacum* L.  (Solanaceae)  SMH-768 | Y | N | N | Tamokh  Tobacco (J) | Aerial Part | Paste | -------------------- | Aerial part is made into paste and applied topically to get rid from maggots. | Bakarwal  Pahari  Dogra |
| *Noccaea caerulescens* (J. Presl & C. Presl)  (Brassicaceae)  SMH-769 | N | N | Y | Bumbuk  Bre-g (L) | Seeds | Powder | -------------------- | Seeds are sun dried, Powdered, mixed with water and salt given twice a day to treat urine incontinence. | Brokapa  Balti |
| *Oryza sativa* L.  (Poaceae)  SMH-770 | Y | Y | N | Daayain gaas (K)  Paraal (J) | Seed husk  Aerial part | Raw  Paste | Raw aerial part is used as such and seed husk is mixed with water to form paste like and used as fodder. The said paste in Kashmir is known as *POI* and *TOOSHI* in Jammu*.* | -------------------- | Kashmiri  Pahari |
| *Oxalis corniculata* L.  (Oxalidaceae)  SMH-771 | Y | N | N | Khatti booti (J) | Whole plant | Raw | Raw whole plant is given as fodder. | -------------------- | Dogra |
| *Parrotiopsis jacquemontiana* (Decne.) Rehder  (Hamamelidaceae)  SMH-772 | Y | Y | N | Kani (J)  Pohu (K) | Leaves | Raw | Raw leaves are given as fodder. | -------------------- | Bakarwal  Pahari |
| *Pedicularis longiflora* Rudolph  (Orobanchaceae)  SMH-166 | N | N | Y | Lugru  Luguruk  Serpo (L) | Flowers | Powder | -------------------- | Flowers are shade dried, powdered given with Luke warm water twice a day to treat kidney issues. | Brokapa |
| *Peganum harmala* L.  (Nitrariaceae)  SMH-773 | N | Y | Y | Sepan,  Harmal (L)  Isband (K) | Seeds | Powder | -------------------- | Obtained powder is given with water to treat liver disorders. | Kashmiri  Dogra  Brokapa |
| *Persicaria hydropiper* (L.) Delarbre  (Polygonaceae)  SMH-774 | Y | Y | Y | Marchwangan (K)  Pipla (J)  Jal mirchi (L) | Leaves | Paste | -------------------- | Leaves are made into paste and applied to the tongue to treat tongue infections. | Kashmiri  Dogra  Brokapa |
| *Plantago depressa* Willd.  (Plantaginaceae)  SMH-140 | N | N | Y | Tha-ram (L) | Leaves | Raw | Raw leaves are used as fodder. | -------------------- | Brokapa |
| *Plantago lanceolata* L.  (Plantaginaceae)  SMH-478 | Y | Y | Y | Isabgol (J)  Gul (K)  Go-lan-see-oh (L) | Seed  Leaves | Raw  Paste | Raw leaves are used as fodder. | Seeds are powdered mixed with water to form a paste which is given orally to treat endoparasites and stomach problems. | Kashmiri  Dogra  Pahari  Brokapa  Balti |
| *Plantago major* L  (Plantaginaceae)  SMH-479 | Y | Y | Y | Tharam (L)  Kakrache (J)  Zimbrejhar (J)  Bud Gulla (K) | Leaves | Raw | Raw leaves are used as fodder. | -------------------- | Kashmiri  Dogra  Pahari  Brokapa  Balti |
| *Podophyllum hexandrum* Royle  (Berberidaceae)  SMH-481 | N | N | Y | Demokusu Papra  Ol-mose (L) | Fruits | Raw | -------------------- | Raw fruits are given orally to smoothen urine discharge. | Brokapa  Balti |
| *Polygonatum verticillatum* (L.) All.  (Asparagaceae)  SMH-775 | Y | Y | Y | Ra-mnye (L)  Kutki (J)  Seal (K) | Rhizome | Powder | -------------------- | Rhizome is sun dried and powdered, given with water orally to treat diarrhoea. | Kashmiri  Dogra  Pahari  Balti |
| *Populus alba* L.  (Salicaceae)  SMH-776 | Y | Y | Y | Bren (L)  Saphdayda (J)  Frass (K)  Kashur-phras (K) | Leaves | Raw | Raw leaves are used as fodder. | -------------------- | Kashmiri  Dogra  Pahari  Brokapa  Balti |
| *Prangos pabularia* Lindl.  (Apiaceae)  SMH-777 | N | N | Y | Prangs  Palano  Prangos  Plans (L) | Roots | Powder | -------------------- | Powder is mixed with water and given at night orally to treat constipation. | Brokapa  Balti |
| *Prunus armeniaca* L.  (Rosaceae)  SMH-340 | Y | Y | Y | Phaling (L)  Charooti (J)  Khubani (J,K)  Chair (K) | Seeds | Oil | Oil obtained from the seed is applied on the hair of goats to increase growth. | -------------------- | Kashmiri  Bakarwal  Pahari  Dogra  Brokapa  Balti |
| *Quercus leucotrichophora* A. Camus  (Fagaceae)  SMH-778 | Y | N | N | Banj oak (J) | Leaves | Raw | Raw leaves are given as fodder. | -------------------- | Pahari  Bakarwal |
| *Ranunculus bulbosus* L.  (Ranunculaceae)  SMH-779 | Y | N | N | Maleen (J) | Roots | Powder | -------------------- | To cure Pneumonia and intestinal worms, the roots are dried, powdered in water and administered orally. | Pahari  Bakarwal |
| *Raphanus sativus* L.  (Brassicaceae)  SMH-780 | Y | Y | Y | Labook (J)  Mooli (K) | Roots | Tea | -------------------- | Tea obtained from fresh roots is given empty stomach to overcome body heat. | Bakarwal  Kashmiri  Dogra  Pahari  Brokapa  Balti |
| *Rhododendron campanulatum* D. Don  (Ericaceae)  SMH-781 | N | Y | Y | Inega (L)  Tama (L)  Tajagsena (K)  Kashmiri Patha (K)  Gagga (J, K) | Flowers | Crushed | -------------------- | Flowers are crushed and then given to animal orally to treat infertility. | Bakarwal  Brokapa  Balti |
| *Rubia cordifolia* L.  (Rubiaceae)  SMH-168 | Y | Y | Y | Btsod (L)  Manjit (J)  Majeeth (J)  Rubes (K) | Roots | Powder | -------------------- | Roots are Powdered, mixed with water and given twice a day to treat inflammation of nipples. | Bakarwal  Kashmiri  Pahari  Brokapa  Balti |
| *Rubus ellipticus* Sm.  (Rosaceae)  SMH-349 | Y | Y | N | Aakhra (J,K)  Aakhey (J,K)  Hinsalu (J,K) | Fruits  Roots  Leaves | Raw | Raw leaves are used as fodder. | Raw fruits and roots are used to treat constipation. | Bakarwal  Kashmiri  Pahari |
| *Rumex hastatus* D. Don  (Polygonaceae)  SMH-351) | Y | Y | N | Khatimal (J,K) | Leaves  Root | Raw | Raw leaves are used as fodder.  Roots are tied to the any part of the body to overcome black magic. | -------------------- | Bakarwal  Kashmiri  Pahari |
| *Rumex nepalensis* Spreng.  (Polygonaceae)  SMH-492 | Y | Y | N | Abuj (K)  Abujiii (J,K)  Hula –halfali (J,K) | Leaves  Root | Raw | Raw leaves are used as fodder.  Roots are tied to the any part of the body to overcome black magic and weakness. | -------------------- | Bakarwal  Kashmiri  Pahari |
| *Salix alba* L.  (Salicaceae) | Y | Y | N | Yeer pan (K)  Bias (J,K) | Leaves | Raw | Raw leaves are used as fodder. | -------------------- | Kashmiri  Dogra  Pahari |
| *Salix pycnostachya* Andersson.  (Salicaceae)  SMH-206 | N | N | Y | Malchang (L) | Leaves | Raw | Raw leaves are used as fodder. | -------------------- | Brokapa  Balti |
| *Salvia abrotanoides* (Kar.) Sytsma  (Lamiaceae)  SMH-782 | N | N | Y | Iskiling  Tarobu (L) | Roots | Poultice | -------------------- | Poultice is made by mixing crushed roots with sesame oil and applied topically to treat skin problems. | Brokapa  Balti |
| *Sambucus wightiana* Wall. ex Wight &Arn.  (Adoxaceae)  SMH-495 | N | Y | N | Ganullo  Faqual (K) | Leaves  Stem | Extract | -------------------- | Fresh leaves and stem are extracted. The said extract is applied for wound healing. | Bakarwal  Kashmiri  Pahari |
| *Sapindus saponaria* Lour.  (Sapindaceae)  SMH-783 | Y | N | N | Rintha (J) | Seeds | Powder | -------------------- | Dried seeds are grinded to powder which is applied to nostrils for the removal of nasal worms. | Bakarwal  Dogra  Pahari |
| *Sarcococca saligna* (D. Don) Müll.-Arg.  (Buxaceae)  SMH-784 | Y | N | N | Duon (J) | Leaves | Paste | -------------------- | Leaves are chopped and made into paste and applied in night to get rid from maggots. | Dogra  Pahari |
| *Saussurea gnaphalodes* (Royle ex Royle) Sch. Bip.  (Asteraceae)  SMH-785 | N | N | Y | Yuliang (L) | Whole plant | Powder | -------------------- | Whole plant is powdered, dissolved in Luke warm water and given orally to treat painful urination. | Brokapa  Balti |
| *Saussurea taraxacifolia* (Lindl. ex Royle) Wall. ex DC.  (Asteraceae)  SMH-786 | N | N | Y | Psangijarpachan  Spangsea (L) | Rhizome | Powder | -------------------- | Sun dried rhizome is powdered, dissolved in Luke warm water and used after three days to treat fever. | Brokapa  Balti |
| *Saxifraga flagellaris* Willd.  (Saxifragaceae)  SMH-787 | N | N | Y | Teetasarzing  Sumchutik (L) | Aerial Part | Paste | -------------------- | For cuts and wounds raw aerial part is made into paste (grinding in traditional stone made pot), applied topically. | Brokapa  Balti |
| *Scutellaria heydei* Hook. f.  (Lamiaceae)  SMH-788 | N | N | Y | Jimthiglae (L) | Aerial Part | Paste | -------------------- | Raw aerial part is made into paste and applied on eyelids and around to treat eye pain. | Brokapa  Balti |
| *Senna tora* (L.) Roxb.  (Fabaceae)  SMH-789 | Y | N | N | Panwar (J) | Leaves | Raw | Raw leaves are used as fodder and to increase milk yield. | -------------------- | Bakarwal  Dogra |
| *Solanum nigrum* L.  (Solanaceae)  SMH-354 | Y | Y | Y | Vari pumble (K)  Tsigma (L)  Kach mach (J)  Kumb kual  Kambii (J,K)  Kach-mach (J,K) | Fruit  whole plant | Raw | Raw Fruits are given to increase body weight and milk production. | -------------------- | Kashmiri  Dogra  Pahari  Brokapa  Balti |
| *Spinacia oleracea* L.  (Amaranthaceae)  SMH-790 | N | N | Y | Vilayati palak (L) | Leaves | Raw | Oxen are given fresh raw leaves followed by luke warm water to gain strength. | -------------------- | Brokapa  Balti |
| *Taraxacum officinale* (L.) Weber exF.H.Wigg.  (Asteraceae)  SMH-504 | Y | Y | Y | Hand (K)  Bathur (J,K)  Phul Dudhli (J,K)  Khur-mang (L) | Roots | Decoction | -------------------- | Decoction obtained from the dried roots is given twice a day for a week to ease delivery. | Kashmiri  Dogra  Pahari  Brokapa  Balti |
| *Thymus linearis* Benth.  (Lamiaceae)  SMH-506 | N | Y | Y | Jaween (K)  Jangli Javind (K)  Tumburu (L) | Seeds | Roasted | -------------------- | Seeds are roasted and given to treat diarrhoea. | Bakarwal  Pahari  Brokapa  Balti |
| *Thymus serpyllum* L.  (Lamiaceae)  SMH-791 | Y | Y | Y | Banajwain (J)  Ajwan (J,K)  Jangli Javind (K)  Tumba (L)  Tumbrak (L) | Seeds | Raw | Seeds are generally given with *gur* during the winters to generate heat in winters. | -------------------- | Bakarwal  Brokapa  Balti |
| *Triticum aestivum L.*  (Poaceae)  SMH-800 | Y | N | Y | Kro (L)  Kanak, Nadhi (J) | Seeds  Whole plant | Powder | Plants are dried and kept for winter usage (fodder). When straw mixed with the *Hordeum vulgare* it form unique combination called as *PHUNGMA* in Ladakh and *POAOO* in Jammu. | ------------------- | Kashmiri  Dogra  Pahari  Brokapa  Balti |
| *Trifolium alexandrinum* L.  (Fabaceae)  SMH-792 | Y | Y | N | Barseem (J)  Bersim (J,K) | Whole plant | Raw | whole plant used as fodder, it is also a constituent species of unique fodder preparation called as *LOV* in Kashmir*,* in Jammu it is constituent species of feed preparation known as *KARB.* | -------------------- | Kashmiri  Dogra  Pahari |
| *Trifolium fragiferum* L.  (Fabaceae)  SMH-793 | N | Y | N | Gaas (K) | Aerial part | Raw | Raw leaves are used as fodder to increase milk yield. In Kashmir, it is also a constituent species of unique fodder preparation called as *LOV*. | -------------------- | Kashmiri |
| *Trifolium pratense* L.  (Fabaceae) | Y | Y | N | Khanda posh (K)  Lalchopati (J)  Biken Boti (J) | Leaves | Raw | Raw leaves are used as fodder to increase milk yield. It is also a constituent species of unique fodder preparation in Kashmir called as *LOV*, it is also a constituent species of fodder preparation in Jammu called *KARB.* | -------------------- | Kashmiri  Dogra  Pahari |
| *Trifolium repens* L  (Fabaceae)  SMH-508 | Y | Y | N | Khanda posh (K)  Boti (J) | Leaves | Raw | Raw leaves are used as fodder to increase milk yield. It is also a constituent species of unique fodder preparation in Kashmir called as *LOV*, it is also a constituent species of fodder preparation in Jammu called *KARB.* | -------------------- | Kashmiri  Dogra |
| *Trifolium resupinatum* L.  (Fabaceae)  SMH-794 | Y | N | N | Shatala (J) | Whole plant | Raw | Raw whole plant used as fodder. It is also a constituent species for a unique fodder preparation in Jammu called *KARB.* | -------------------- | Pahari |
| *Ulmus wallichiana* Planch  (Ulmaceae)  SMH-795 | N | Y | N | Bran (K) | Leaves  Twig | Raw | Raw leaves are used as fodder. Twig is tied to the animal especially to leg to overcome evil spirit problems. | -------------------- | Kashmiri  Pahari |
| *Urtica dioica* L.  (Urticaceae)  SMH-510 | N | N | Y | Rdasot (L) | Roots | Powder | Roots are crushed and powdered and given to cattle orally to increase milk production. | -------------------- | Brokapa  Balti |
| *Urtica hyperborea* Jacq. ex Wedd.  (Urticaceae)  SMH-171 | N | N | Y | Dzatsutt  Zahchot  Zasot (L) | Leaves | Powder | -------------------- | Leaves are dried, powdered and given with water for Y0 consecutive days to treat stomach disorders. | Brokapa  Balti |
| *Verbascum thapsus* L  (Scrophulariaceae)  SMH-511 | Y | Y | Y | Giddhartamaku (J)  Gidat tamakoo (J)  Jungli tamook (K)  Ngo-serjee (L) | Aerial Part  Rhizome | Raw  Decoction | -------------------- | Raw fresh aerial part is given twice a day to treat loose motion. Decoction obtained from the rhizome is boiled in water and given orally to treat Pyrexia, and Infertility. | Kashmiri  Pahari  Brokapa  Balti |
| *Viburnum grandiflorum* Wall. ex DC.  (Adoxaceae)  SMH-358 | Y | Y | N | Kulmosh (K)  Kulmanch (J)  Kilmunch (J)  Chalandar (J)  Kulmaach (K) Gouch (K) | Leaves | Raw | Raw leaves are used as fodder. | Raw leaves are used to treat constipation. | Bakarwal  Kashmiri  Pahari |
| *Vicia hirsuta* (L.) Gray  (Fabaceae)  SMH-796 | Y | N | N | Papda (J) | Whole plant | Raw | Raw whole plant is used as fodder. | -------------------- | Pahari  Dogra |
| *Vicia sativa* L.  (Fabaceae)  SMH-797 | Y | N | N | Phalli (J) | Whole plant | Raw | Raw whole plant is used as fodder. | -------------------- | Pahari  Dogra |
| *Vitex negundo* L.  (Lamiaceae)  SMH-798 | Y | N | N | Banna (J) | Leaves  Root | Raw | Roots are made into amulet to overcome evil eye. | Raw leaves are boiled for Y5 minutes in 2 litres of water and given to adult ruminants orally twice a day to treat cold, cough, and worm problems. | Bakarwal  Dogra |
| *Withania somnifera* (L.) Dunal  (Solanaceae)  SMH-799 | Y | N | N | Ashwagandha  Asgandh (J) | Whole plant | Powder | -------------------- | Whole plant is dried, powdered and given orally with water to treat sexual disorders, ulcers and burns in cats and other pets. | Bakarwal |
| *Zanthoxylum armatum* DC.  (Rutaceae)  SMH-515 | Y | N | N | Timbru  Timber  Timru  Temer (J) | Seeds  Fruit  Leaves | Decoction | -------------------- | Decoction obtained from seeds, fruits and leaves is given orally to treat cold, fever and body pain. | Bakarwal |
| *Zea mays* L.  (Poaceae)  SMH-605 | Y | Y | Y | Makai, Maki-wat (K)  Makka (L)  Mak (J) | Seeds  Whole plant | Powder | Plants are dried and kept for winter usage (fodder), locally in Kashmir it is called as *Kaand,* in Jammu it is called as *Makard*. | Seeds are grinded into flour which is made into bread and given to animal to treat gastro intestinal disorders. | Kashmiri  Dogra  Pahari  Brokapa  Balti |
| *Ziziphus mauritiana* Lam.  (Rhamnaceae)  SMH-606 | Y | N | N | Ber (J) | Leaves | Raw | Raw leaves are given as fodder. | Raw leaves are given to treat Dysentery. | Bakarwal  Dogra |
